# *In vivo* screen for osteoclast derived osteogenic factors using non-resorptive osteoclasts in zebrafish bone

**DOI:** 10.1093/g3journal/jkag221

**Published:** 2026-08-20

**Authors:** Joana Caetano-Lopes, Valentina Daponte, Katia Urso, Rachel L Roberts, Matthew L Warman, Hicham Drissi, Julia F Charles, Katrin Henke

**Affiliations:** Orthopedic Research Laboratories, Boston Children's Hospital, Boston, MA 02115, United States; Department of Genetics, Harvard Medical School, Boston, MA 02115, United States; Department of Orthopaedics, Emory University School of Medicine, Atlanta, GA 30329, United States; VA Research, VA Medical Center, Atlanta, GA 30033, United States; Departments of Orthopaedics and Medicine, Brigham and Women's Hospital and Harvard Medical School, Boston, MA 02115, United States; Department of Orthopaedics, Emory University School of Medicine, Atlanta, GA 30329, United States; Orthopedic Research Laboratories, Boston Children's Hospital, Boston, MA 02115, United States; Department of Genetics, Harvard Medical School, Boston, MA 02115, United States; Department of Orthopaedics, Emory University School of Medicine, Atlanta, GA 30329, United States; VA Research, VA Medical Center, Atlanta, GA 30033, United States; Departments of Orthopaedics and Medicine, Brigham and Women's Hospital and Harvard Medical School, Boston, MA 02115, United States; Department of Orthopaedics, Emory University School of Medicine, Atlanta, GA 30329, United States

**Keywords:** zebrafish, osteopetrosis, *clcn7*, CLC-7, osteoclast, anabolic, screen

## Abstract

Bone homeostasis requires the balanced action of bone forming osteoblasts and bone resorbing osteoclasts. This balance is maintained through local as well as systemic factors. Loss of osteoclast resorptive activity leads to development of osteopetrosis with increased bone volume but reduced skeletal integrity. Identification of the factors that regulate such anabolic activity is hampered by the essential function of these genes in development as well as the inaccessibility of large numbers of primary osteoclasts for biochemical analyses. Here, we generated zebrafish with nonresorptive osteoclasts through CRISPR-Cas9 mediated knockout of *chloride voltage-gated channel 7* (*clcn7*). The *clcn7* mutants are adult viable, show increased vertebral bone thickness and increased bone formation. Using this *in vivo* model of persistent, but deficient osteoclasts, we used differential gene expression to identify putative anabolic bone regulators. Transcriptional profiling of *clcn7* mutant primary osteoclasts identified differential expression of genes involved in chemotaxis and cytokine signaling. Global transgenic overexpression of a subset of the upregulated, secreted genes showed positive effects of the cytokines *csf3b, cxcl19*, and the glycosylated peptide *progranulin 1* on skeletal parameters, while the cytokine *ccl35.1* negatively impacted bone volume. Our findings suggest that the concerted function of these cytokines plays an important role in the regulation of bone homeostasis and that individual cytokines cause distinct changes in vertebral bone form and differentiation.

## Introduction

Despite its static appearance, the skeleton is a dynamic structure that is constantly remodeled throughout life by the coupled resorption of bone by osteoclasts and formation by osteoblasts (Bolamperti et al. 2022). Remodeling of bone is essential to maintain structural integrity of the skeleton through removal of old or damaged tissue and in response to changes in mechanical load. The remodeling process further maintains a crucial physiological balance of systemic calcium and phosphate levels through their release from, or incorporation into the bone matrix. To achieve these regulatory functions, bone catabolism and anabolism are tightly regulated by local and systemic factors that coordinate and balance the action of osteoclasts and osteoblasts. This regulation of bone and mineral content with functional and physiological demand is a lifelong process, and its deregulation is a common source of age-related pathologies, leading to osteoporosis and increased fracture burden in older individuals.

Secreted signaling molecules mediate bone cell communication, several of which have been identified. Osteoblast secreted factors, such as RANKL and OPG, regulate osteoclast formation and resorptive activity, while sclerostin released by osteocytes regulates anabolic osteoblastic activity by inhibiting canonical WNT signaling (Boyce and Xing 2008; Marini et al. 2023). In contrast, the regulation of osteoblasts by osteoclasts is less well understood. Proposed coupling mechanisms between these cell types include the release of matrix stored growth factors (TGF-β, IGF-1) through osteoclastic resorption and the secretion of soluble factors termed “clastokines” from osteoclasts (Daponte et al. 2024).

One approach for identifying clastokines and studying their effect on osteoblasts is by studying diseases affecting the skeleton that are caused by osteoclast dysfunction. Osteopetrosis is an umbrella term for a group of rare genetic disorders characterized by defective bone resorption leading to an increase in bone density and volume but also a concomitant decrease in bone mechanical properties (Funck-Brentano et al. 2024). There are distinct phenotypic differences between disease that affect the function of osteoclasts (osteoclast-rich osteopetrosis) and those that decrease overall number (osteoclast-poor osteopetrosis). A comparison of the skeletal phenotype between these 2 conditions shows greater bone formation in the presence of nonfunctional osteoclasts than when they are absent (Thudium et al. 2014). This supports a model for clastokines in which osteoclasts are actively instructing osteoblast activity via the release of clastokines.

One gene associated with osteoclast-rich osteopetrosis is *CLCN7*, encoding the chloride voltage-gated channel 7 (CLC-7). CLC-7 exchanges chloride ions and protons, and together with the vacuolar ATPase (V-ATPase), is essential for the acidification of resorption lacunae and thus degradation and resorption of the bone matrix (Zifarelli 2022). Mice with loss of CLC-7 function develop severe osteopetrosis due to impaired bone resorption (Kornak et al. 2001). The resulting inability to form a marrow cavity leads to bone marrow failure and death before 8 wk of age. The early death of *Clcn7* knockout mice before reaching skeletal maturity hinders broad investigation into the roles of clastokines produced by *Clcn7* mutant osteoclasts. Another limiting factor for studying clastokines in mice is the difficulty of recovering them from skeletal tissue due to their scarcity and strong adhesion to bone. Therefore, investigations into clastokines have focused on *in vitro* studies, using conditioned medium from *in vitro* differentiated osteoclasts to identify differentially expressed factors. These *in vitro* tools are powerful but lack the complex environment that affects osteoclast differentiation and function *in vivo*.

Small teleost fish, such as zebrafish, have increasingly been used to study skeletal development and disease. This is in part due to the high level of conservation of genetic regulation of skeletal development among vertebrates, as well as the high fecundity and ease of genetic manipulation (Busse et al. 2020). Specific to osteoclast biology, the small size of zebrafish and the existence of transgenic lines which express fluorescent proteins in osteoclast-lineage cells facilitate the recovery of primary osteoclasts from skeletal tissues by fluorescent activated cell sorting (FACS) (Buettner et al. 2018; Caetano-Lopes et al. 2020). Finally, in contrast to mice and humans, who do not survive as adults without functioning osteoclasts, fish lacking resorbing osteoclasts are viable and fertile (Caetano-Lopes et al. 2020).

Here, we used adult zebrafish with a *clcn7* loss-of-function allele, *clcn7^mh109^* (referred to as *clcn7^−^*) to isolate nonresorptive osteoclasts and identify potential clastokines, by comparing osteoclast transcriptomes. We then demonstrate ease of testing candidates through transgenesis, overexpressing the most significantly upregulated potential clastokines to assess the effect on bone homeostasis *in vivo*.

## Materials and methods

### Zebrafish husbandry and strains used

This study was approved by the Institutional Animal Care and Use Committees at Boston Children's Hospital and Emory University (IACUC protocol 13-08-2470R and PROTO202000150, respectively). The animals included in this study were maintained under conditions described in the environmental summary (Lawrence et al. 2021). The following lines were used in this study: *tg(ctsk:DsRed)^mh201^* (Caetano-Lopes et al. 2020), *clcn7^mh109^* (this study), and overexpression lines *ubb:cxcl19, ubb:ccl35.1, ubb:M17, ubb:csf3b, ubb:grn1, ubb:lgals9l* generated in this study. Transgenic and mutant lines were maintained in a mixed Tuebingen/T5D background.

### CRISPR/Cas9 mutagenesis

Cas9 mutagenesis was performed following the protocol described by Jao et al. (2013). Cas9 mRNA was synthesized from the pCS2-nCas9n vector through *in vitro* transcription using the mMESSAGE mMACHINE SP6 kit (Life Technologies). The guide target sequence in exon 2 of *clcn7* was identified using ZiFiT Targeter (Hwang et al. 2013). Oligonucleotides complementary to the target sequence (zb-gRNA FW 5′-TAGGGGGAGGTATATTTCATAT-3′; RV 5′-AAACATATGAAATATACCTCCC-3′) were annealed and cloned downstream of the T7 promoter into the pT7-gRNA vector. The linearized gRNA vector was then transcribed *in vitro* to produce gRNA using the MEGAShortscript T7 kit (Life Technologies). The pCS2-nCas9n and pT7-gRNA plasmids were a gift from Wenbiao Chen (Addgene plasmid # 47929; http://n2t.net/addgene:47929; RRID: Addgene_47929 and Addgene plasmid # 46759; http://n2t.net/addgene:46759; RRID: Addgene_46759, respectively).

To generate mutations, a mix of gRNA (25 ng/μl) and Cas9 mRNA (400 ng/μl) was injected into one-cell stage zebrafish embryos. Cutting efficiency was analyzed at 1 d-postfertilization by amplification of a 400 bp genomic region surrounding the target site in exon 2 of *clcn7* using primers *clcn7-g-f* (5′-ttaaggatcacattccagcacca-3′) and *clcn7-g-r* (5′-agctcaaagcaagtactgaaaca-3′) followed by T7 endonuclease digestion. To establish mutant lines, injected fish were grown to adulthood and outcrossed to wild-type (WT) fish. Genomic DNA from F1 progeny was extracted from caudal fins and mutant carriers identified by heteroduplex PCR using the same primers. The nature of the induced mutations was identified through Sanger sequencing of subcloned PCR products.

For identification of fish carrying the *clcn7^mh109^* mutation, which deletes a MwoI restriction site, genomic DNA from caudal fin clips from adult zebrafish was isolated and amplified using primers *clcn7-g-f + clcn7-g-r*. PCR products were digested with MwoI (NEB) and analyzed by gel electrophoresis.

### von Kossa and tartrate-resistant acid phosphatase staining

For von Kossa and tartrate-resistant acid phosphatase (TRAP) staining, we collected scales from the same area (anterior trunk along the lateral midline, between the head of the fish and start of the dorsal/anal fin) of 10-wk-old *clcn7^−/−^* and WT siblings (*n* = 10/genotype).

To analyze bone resorption, a modified von Kossa protocol was used as described before (Caetano-Lopes et al. 2020). Immediately after collection, scales were stained in an aqueous 2.5% silver nitrate solution (Sigma) for 30 min at room temperature, then washed and stored in distilled water. Imaging was performed using a Leica DM2000 microscope. For quantification of bone resorption using ImageJ, a threshold was applied to the image and the “analyze particle” function was used to determine the total scale area as well as the resorbed area (unstained regions within the scale) (Schneider et al. 2012). To account for differences in scale size, the percentage of resorbed area relative to the total scale area was calculated.

To visualize osteoclasts, scales were fixed in 10% formalin, washed twice with distilled water, and incubated with TRAP staining solution (100 μg/ml Naphtol AS-Mx phosphate, 1% *N*,*N*-dimethylformamide, 600 μg/ml Fast Red LB Violet salt in 0.1 M acetate buffer, pH 5.0) at 37 °C for 10 to 15 min. The number of scales with and without positive TRAP signal was counted for each fish, and data are presented as the percentage of positive cells/fish.

For each staining, at least 6 scales per fish were analyzed.

### Micro-computed tomography

Three-months-old fish were euthanized with an overdose of tricaine methanesulfonate (MS-222, 200 to 300 mg/l) solution, fixed in 3.7% formaldehyde/PBS at RT for 24 h and washed and stored in PBS. Micro-computed tomography (micro-CT) scanning at 20 μm voxel resolution was performed on a Scanco μCT 50 with the following settings: 55 kVp, 145 μA, 8 W, Filter AL 0.5 mm, 1,024 slices, and 200 ms integration time. The slope and intercept for the calibration of tissue mineral density (TMD) was determined by density analysis of a phantom scanned using the same parameters. For scanning, fish were rolled into polyurethane foam and placed into a 14 mm diameter sample holder filled with PBS. Scans were converted into Digital Imaging and Communications in Medicine images using the Scanco software and analyzed with the FishCut software using a threshold of 0.73 for all images (Hur et al. 2017). To account for any confounding effects caused by differences in standard length (SL), WT fish were normalized using a previously published allometric scaling method and the associated R scripts (Hur et al. 2017). First, we established a scaling matrix using values obtained from all 70 WT siblings analyzed, which had a range of SL from 22 to 27 mm (Supplementary Table 1). Next, we normalized skeletal parameters of all WT siblings to the average mutant, or overexpression line, length using the scaling matrix as described by Hur et al. (2017).

To summarize the data and visualize differences between WT siblings and *clcn7^−/−^* fish, or overexpression lines, *z*-scores were computed for all outcomes as the difference between the mean value across all vertebrae in the knockout/overexpression line pool and the mean value across all vertebrae in the corresponding WT sibling pool, divided by the standard deviation (SD) across all vertebrae in the WT sibling population.

### *In vivo* bone formation assay

The *in vivo* bone formation assay was performed as described before (Urso et al. 2019). At 7-wk-postfertilization (wpf), progeny from heterozygous *clcn7^+/−^* incrosses were transferred into 100 μg/ml calcein in system water (Sigma-Aldrich) and stained overnight. The next day, fish were rinsed 3 times in system water and kept off system in static tanks with system water for 14 d. Fish were fed according to their regular schedule, and water was changed every other day. After 14 d, fish were transferred to an alizarin red solution (100 μg/ml alizarin red, 10 mM HEPES, pH 7.0, Sigma-Aldrich) and stained overnight. The next day, fish were rinsed 3 times in system water before they were euthanized. Scales were removed from the anterior trunk and imaged using a fluorescence stereomicroscope with long-pass filter. In addition, fin tissue was collected for genotyping. No adverse events were observed during *in vivo* staining.

The area of total bone (alizarin red stains, TB) and pre-existent bone (calcein stain, CB) was measured with ImageJ software (Schneider et al. 2012). The percentage of new bone formed in the 14 d (the area stained with alizarin red only) was calculated with the formula: % new bone formed = 100 × (TB − CB/TB). Six fish per group and six scales per fish were analyzed.

### Cell isolation and sorting

To generate fish for osteoclast cell sorting, heterozygous *cln7^+/−^* fish were crossed with the *tg(ctsk:DsRed)* line to produce *clcn7^+/−^;tg(ctsk:DsRed)* offspring. These fish were then crossed with *clcn7^+/−^* to generate *WT;tg(ctsk;DsRed)* and *clcn7^−/−^;tg(ctsk:DsRed)*. 10 wpf fish were euthanized, and tissue samples were harvested for isolation of cells. Cell isolation and FACS were performed as previously described (Caetano-Lopes et al. 2020). Tissues were digested by vigorous shaking at 30 °C in dissociation solution [0.21% trypsin-EDTA (Gibco), 16 mg/ml collagenase from *Clostridium histolyticum*, type IA (Sigma)] for 15 min. The cell suspension was removed and filtered through a 40 µm nylon mesh cell strainer. Fresh dissociation solution was added, and the remaining tissue was digested by vigorous shaking at 30 °C for 15 min. The cell suspension was washed with Dulbecco’s modified Eagle media supplemented with 10% fetal bovine serum (FBS, Invitrogen) and filtered through a 40 µm nylon mesh. The filtered cell suspension was layered on Histopaque-1077 (Sigma) at a 2:1 ratio and centrifuged at 400 × *g*, without acceleration or break, for 30 min in a swinging bucket rotor. Both the ring and the pellet were collected in PBS + 2% FBS. Immediately prior to sorting, cells were stained with 1× SYTOX Blue (Invitrogen). After doublet exclusion, live cells were identified by the absence of SYTOX Blue staining. *DsRed*-positive cells were separated on an FACSAria II cell sorter (BD Biosciences) using an 85 µm nozzle at a flow rate of ∼2,000 events/s; 5,000 *DsRed*-positive cells per fish were collected, and cells from 2 fish were combined for each biological replicate. Sorted cells were collected directly into RNA lysis buffer, and RNA was extracted using the Qiagen RNeasy Micro kit. Three biological replicates were prepared per genotype. Library preparation and 150 paired-end sequencing was performed at Azenta/Genewiz.

To analyze DNA content, dissociated cells were counted and diluted to 10^6^ cells per 500 µl. Hoechst 33342 (Sigma) was added at a concentration of 10 µg/ml, and cells were incubated for 30 min at 37 °C. Cells were washed and analyzed for *dsRed* and Hoechst expression using a previously established gating strategy (Caetano-Lopes et al. 2020).

### Bioinformatic analysis

Sequence quality of RNA-Seq data were assessed using FastQC (Andrews 2010), and low-quality bases and adapter sequences were trimmed using TrimGalore (Krueger 2012). Trimmed reads were aligned to the GRCz11 version of the zebrafish genome using STAR (Dobin et al. 2013). The sequence of the primary assembly of the zebrafish genome (Danio_rerio.GRCz11.dna.primary) was retrieved from Ensembl (Cunningham et al. 2022). For gene annotations, an updated annotation file was retrieved from the Lawson Lab (Lawson et al. 2020). Transcript counts were generated with HTSeq, and differential gene expression analysis was performed using the DESeq2 option on the iDEP2.01 website (Love et al. 2014; Anders et al. 2015; Ge et al. 2018). We required 5 counts per million reads to be present in at least 1 library, for a gene to be considered expressed. GO term analysis was performed using ShinyGO 0.76 (Ge et al. 2020). GO term plots were generated in GraphPad Prism version 10.0.0 for Mac, GraphPad Software, Boston, MA USA, www.graphpad.com.

For the identification of secreted factors, the lists of human and zebrafish matrisome genes were retrieved from the Matrisome Project website (Hynes and Naba 2012; Nauroy et al. 2018). Zebrafish orthologs for genes that were annotated as secreted in only the human dataset were identified using the DIOPT Ortholog Finder to create a master list of secreted genes (Hu et al. 2011). In addition, a literature search was performed for the top 250 genes that exhibited increased expression in *clcn7^−/−^* osteoclasts to identify novel secreted proteins not annotated in the matrisome project. Subsequently, the Zebrafish Information Network database was queried for expression and phenotypic data of potential secreted factors (Bradford et al. 2022).

### Overexpression analysis of candidate genes

For overexpression analysis, the open reading frames (ORFs) of candidate genes were synthesized and cloned between the NcoI and KpnI sites into the pDestTol2pA2_ubi:GFP vector, replacing the ORF of the GFP. Gene synthesis and cloning were performed at Azenta/Genewiz. The pDestTol2pA2_ubi:EGFP plasmid was a gift from Leonard Zon (Addgene plasmid # 27323 ; http://n2t.net/addgene:27323; RRID: Addgene_27323) (Mosimann et al. 2011). Plasmids were injected together with Tol2 mRNA into T5D WT embryos, and embryos were raised to adulthood. Transgenic founders were identified through random outcrosses of injected fish and genotyping of 8 progeny from each cross for the presence of the transgene. Progeny from crosses that had at least 1 positive embryo were raised, and adults were fin clipped to identify transgenic fish. Transgenic F1s were again outcrossed, and crosses that showed ∼50% of transgenic progeny, suggesting the presence of a single insertion event, were maintained. Three-months-old F3 progeny were used for skeletal phenotyping.

For identification of transgenic fish, genomic DNA from caudal fin clips from adult zebrafish was isolated and amplified using primers listed in Supplementary Table 2.

### Statistical analysis

Comparisons between 2 groups were analyzed using unpaired Student's *t*-test or Mann–Whitney *U* test, depending on the normality distributions evaluated by D’Agostino and Pearson omnibus normality test. A significance level *P* < 0.05 was considered significant. All statistical analyses were performed with GraphPad Prism 10.0.0 software. Error bars represent SD of biological replicates, unless otherwise indicated.

To assess significant differences in skeletal parameters between groups, the global test package in R was used (Goeman et al. 2004, 2006; Hur et al. 2017; R Core Team 2021).

## Results

We generated non-resorbing osteoclasts to identify undescribed anabolic factors released by osteoclasts. We engineered a *clcn7* mutation in zebrafish (*clcn7^−^*), which is an 8 bp deletion (c.222_229del) in exon 2. This mutation is predicted to cause a frame shift and an early truncation of the protein deleting all functional domains (p.H43Qfs8X, Fig. 1a and b). Progeny from *clcn7^+/−^* incrosses showed expected Mendelian frequencies, and *clcn7^−/−^* fish were viable, fertile and did not show any overt morphological changes (Fig. 1c).

**Fig. 1.**
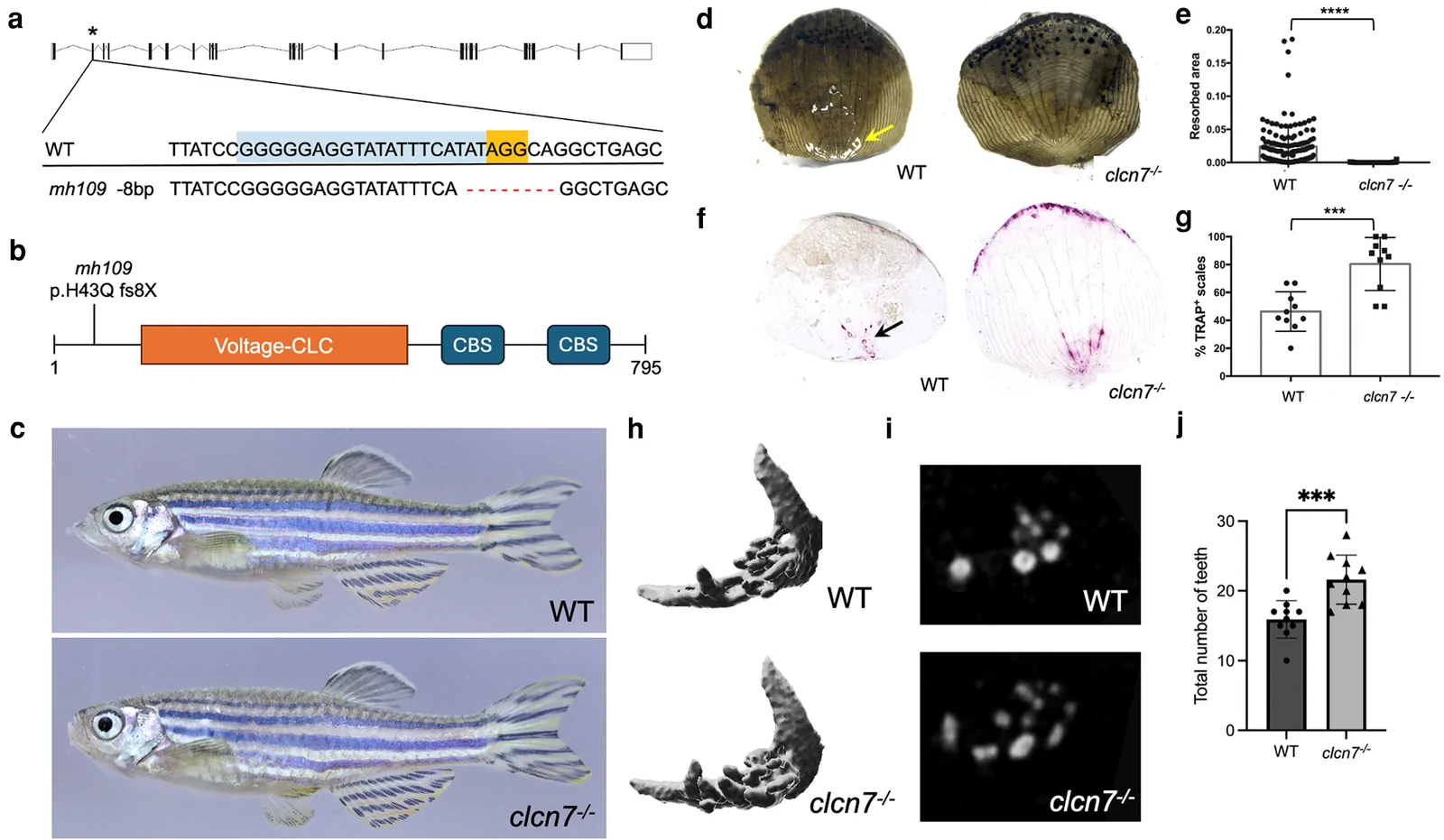
Generation and phenotypes of *clcn7* mutant zebrafish. a) Schematic representation of *clcn7* gene structure on chromosome 3, indicating the guide RNA target site in exon 2 (asterisk) with sequence of WT and the *mh109* mutant *clcn7* allele at the guide target site (blue box) showing an 8 bp deletion in *mh109* predicted to lead to a frameshift and early truncation of the protein. Yellow, PAM sequence. b) Cartoon of the ClC-7 protein sequence showing the predicted consequence of the deletion leading to a frameshift at amino acid 43 and early truncation of the protein (p.H43Qfs8X). Orange box, voltage gated chloride channel domain (voltage-CLC); blue box, protein domain found in cystathionine beta-synthase (CBS), which binds ligands with adenosyl group. c) Homozygous *clcn7* mutants do not show any overt morphological phenotypes. Representative photomicrographs of adult WT (top) and homozygous *clcn7*^−/−^ mutant zebrafish (bottom). d) Representative scales collected from 10 wpf WT and *clcn7^−/−^* zebrafish stained with von Kossa. Arrow in WT indicates the area resorbed by osteoclasts. e) Quantification of the size of the resorbed area in WT and mutants. Each data point represents a scale; 20 scales/fish; *n* = 10 fish. f) Scales of WT and mutant zebrafish stained with TRAP. Arrow in WT indicates TRAP positive area. g) Quantification of the percentage of TRAP positive scales found in WT and *clcn7^−/−^* fish. Each data point represents 1 fish; *n* = 10/group. h) Top view h) and optical section i) through a surface rendering of the pharyngeal arch of 3 mpf WT and *clcn7^−/−^* fish imaged by micro-CT. j) Quantification of the total number of teeth on both pharyngeal arches. *n* = 10/group; **** *P* < 0.0001, *** *P* < 0.001.

We assayed osteoclast number and resorptive activity on scales of WT and *clcn7^−/−^* fish through analysis of TRAP activity and von Kossa staining of mineralized tissue, respectively. Scales were chosen as model structures to analyze osteoclast activity as they are numerous, easy to isolate, process, and image. More importantly, their mineralized collagen matrix is remodeled and populated by osteoclasts and osteoblasts (Metz et al. 2012). Quantification of bone resorption, determined by the unstained areas of matrix localized at the central focus of the scale, revealed near complete absence of resorption in *clcn7^−/−^* mutants compared with WT fish (Fig. 1d and e). Interestingly, TRAP staining showed significantly more *clcn7^−/−^* scales containing osteoclasts compared with WT scales (80% vs 46%, respectively) (Fig. 1f and g). Thus, *clcn7^−/−^* fish have reduced capacity to resorb bone, even though they seem to increase TRAP osteoclast activity.

To assess the phenotypic consequences of defective bone resorption, we visualized skeletons of young adult (3-mo-old) WT and *clcn7^−/−^* fish. One phenotypic consequence of reduced osteoclast numbers can be observed in the remodeling of bone tissue in the dentition, leading to lack of tooth shedding and increased tooth numbers (Caetano-Lopes et al. 2020). Similarly, in the case of *clcn7^−/−^* fish that have osteoclasts that cannot resorb bone, we observed significantly more pharyngeal teeth in *clcn7^−/−^* fish compared with WT (21 vs 16, respectively) (Fig. 1h to j). Using micro-CT, we measured changes in bone volume, thickness, and TMD in abdominal and caudal vertebrae (22 vertebrae total per fish). Because osteoclasts accumulate around the haemal and neural arches of vertebrae in zebrafish (Caetano-Lopes et al. 2020), in addition to examining whole vertebra, we also separately evaluated vertebral centra, neural arches, and haemal arches. For these studies, we focused on broad changes in skeletal morphology reflective in whole structure measures as these will be more reflective of systemic changes affecting the skeleton. Looking across the vertebrae, we found that vertebral thickness was significantly increased (Vert. Th, *P* = 0.001) in *clcn7^−/−^* mutants in all 3 vertebral elements. Additionally, neural arches of *clcn7^−/−^* had significantly increased volume (Neur. Vol, *P* = 0.003) and TMD (Neur. TMD, *P* = 0.016) (Fig. 2).

**Fig. 2.**
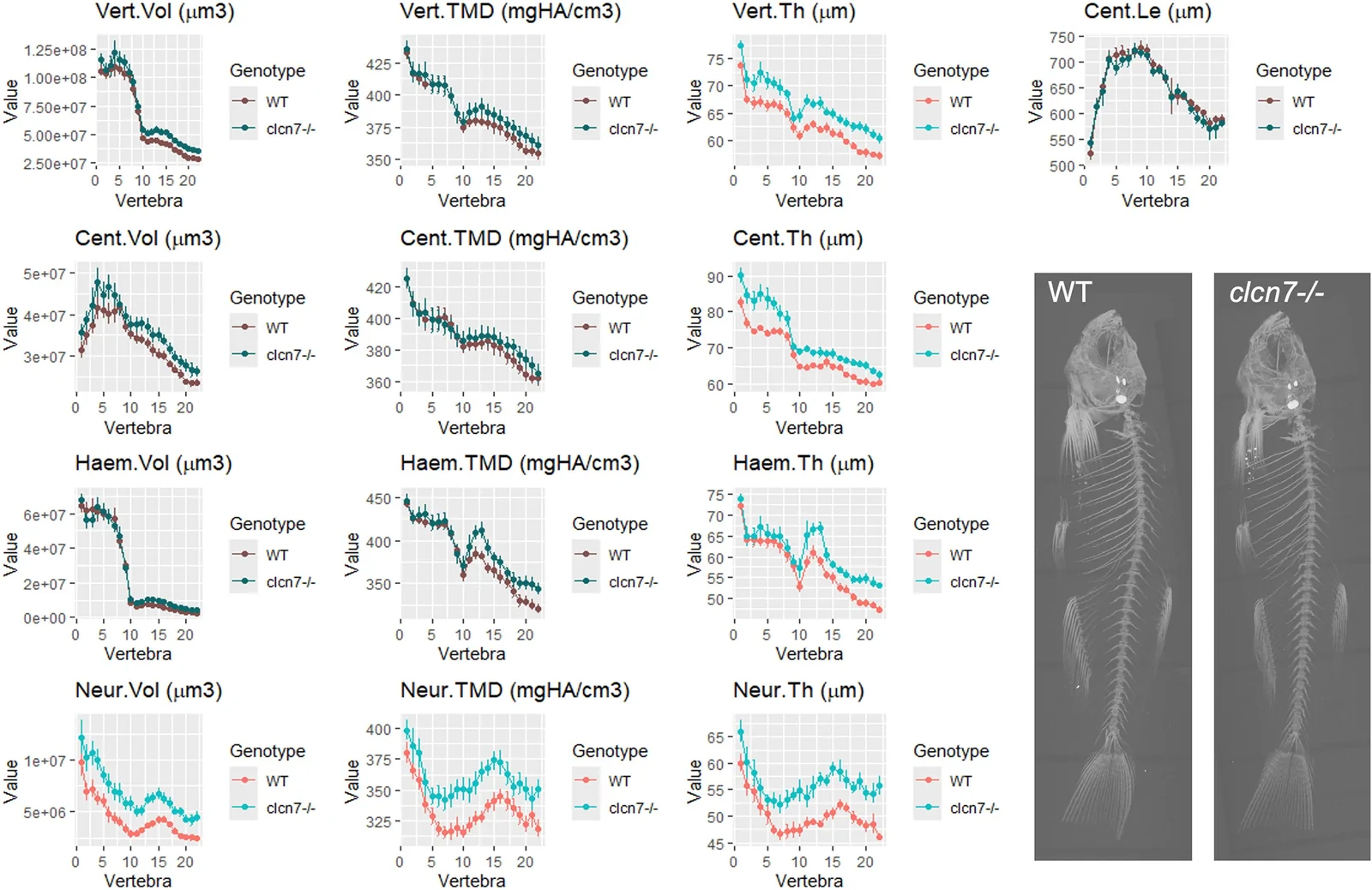
Loss of CLC-7 function increases vertebral thickness as well as volume and TMD of the neural arches. Micro-CT analysis of the vertebral column of 3 mpf WT and *clcn7 ^−/−^* mutants. The volume (Vol), TMD, and thickness (Th) of the whole vertebra (Vert.) as well as values for the centrum (Cent.), haemal arch (Haem.), and neural arch (Neur.) separately are shown. WT data was normalized using allometric normalization. Values for abdominal and caudal vertebrae (22 total) are plotted as mean ± SEM. Measures that were significantly different between WT and *clcn7^−/−^* fish based on the global test are highlighted using lighter coloring (see text for *P* values). *n* = 10/group. Bottom right corner: representative maximum intensity projection of micro-CT scans from WT and *clcn7^−/−^* fish.

We determined if the increases in bone thickness and volume in *clcn7*^−/−^ fish could be due to increased bone formation by measuring bone accrual in scales over a 14 d interval between 7 and 9 wpf (Fig. 3a). There was significantly more bone growth in *clcn7^−/−^* fish, with 21% new bone formed, compared with 19% observed in WTs, suggesting increased osteoblastic activity in *clcn7^−/−^* (Fig. 3b and c).

**Fig. 3.**
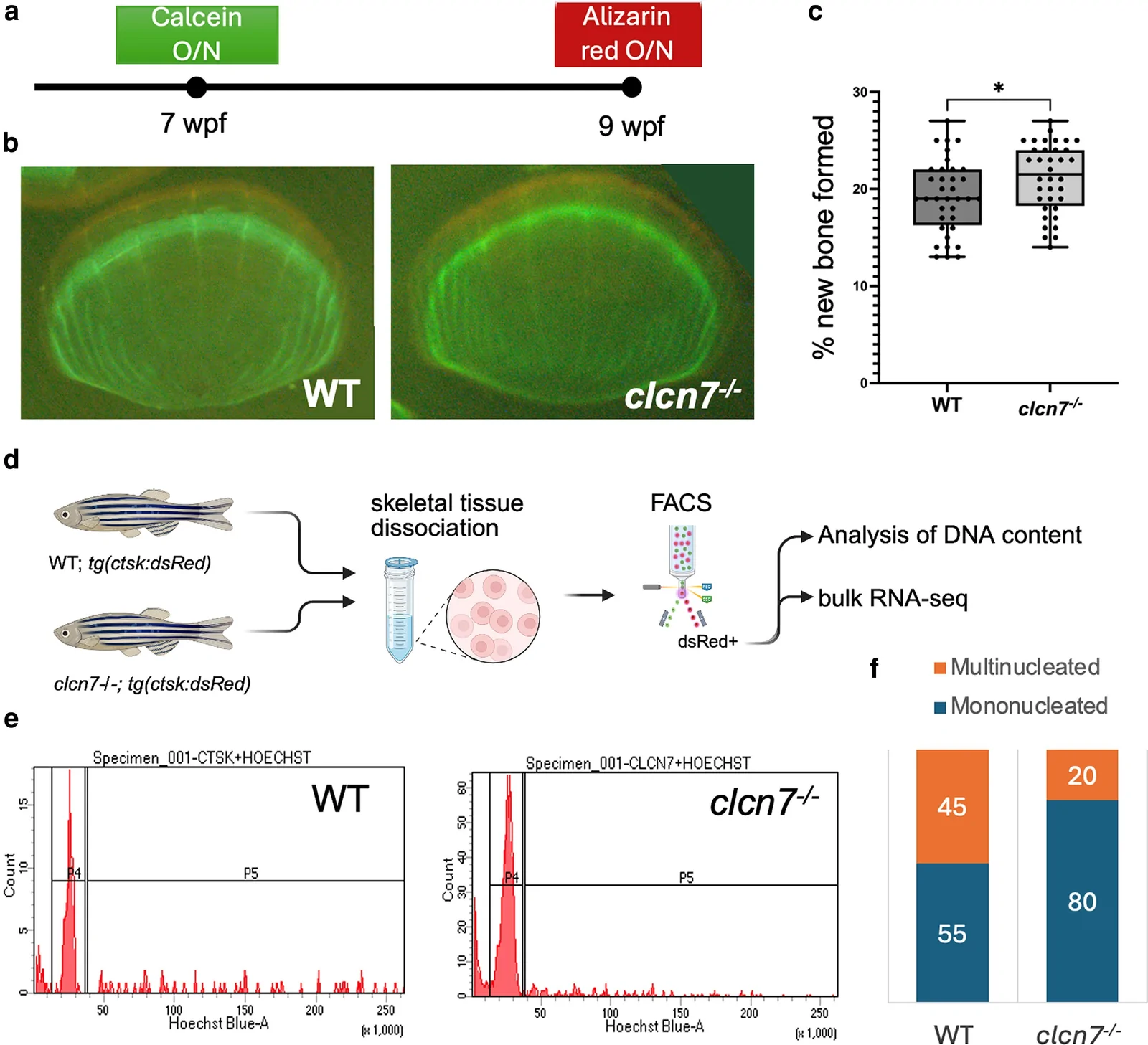
*Clcn7* mutants have increased bone formation and fever multinucleated osteoclasts. a) To label forming bones, fish were stained at 7 wpf with calcein over night (O/N), followed by alizarin red staining at 9 wpf. b) Representative images of sequentially stained scales from WT and *clcn7^−/−^* mutants collected at 9 wpf. The green stained bone front outlines bone present at 7 wpf, and the red staining shows the bone front at 9 wpf. c) Quantification of the percentage of newly formed bone in WT and *clcln7^−/−^*. Data points represent individual scales. *n* = 6 fish/group; 6 scales/fish; * *P* < 0.05. d) Strategy for the isolation of primary osteoclasts from WT and *clcn7^−/−^* fish. Skeletal tissue from *ctsk:dsRed* transgenic WT and *clcn7^−/−^* fish was dissociated, and dsRed-positive cells were subsequently isolated for DNA content analysis and bulk RNA-sequencing using FACS (created in BioRender. Henke, K. (2026) https://BioRender.com/d6yds16). e) Histograms for dsRed-positive cell counts in WT (left) and *clcn7^−/−^* (right). Indicated gates were used to differentiate mononucleated and multinucleated cells based on Hoechst intensity. f) Quantification of mono and multinucleated dsRed-positive cells in WT and *clcn7^−/−^* fish.

To identify clastokines that could be responsible for the increased bone formation, we used the ability to isolate osteoclast populations from adult skeletal material in the zebrafish to perform RNA sequencing in the different genetic contexts. Taking advantage of the *tg(ctsk:DsRed)* transgenic line, which labels mature osteoclasts through *cathepsin K* (*ctsk*) driven DsRed expression, we isolated osteoclasts via FACS (Fig. 3d). In zebrafish, both mono- and multinucleated osteoclasts play a role in bone resorption (Witten et al. 2001; Witten and Huysseune 2009), and both cell types are present in FACS sorted dsRed-positive osteoclasts (Caetano-Lopes et al. 2020). DNA content analysis of FACS sorted dsRed-positive osteoclasts recovered from *clcn7^−/−^* fish revealed a higher ratio of mononucleated to multinucleated osteoclasts compared with WT fish (Fig. 3e and f). For each genotype, we collected 3 pools of 10,000 dsRed-positive cells, combining cells from 2 fish per pool. We obtained >30 million reads per library, of which over 90% of reads could be uniquely aligned to the GRCz11 assembly of the zebrafish genome. Visual inspection of *clcn7* transcripts confirmed the mutant pools were homozygous for the 8 bp deletion in exon 2 (Supplementary Fig. 1a). Visualization of splicing events revealed that alternative splicing leads to skipping of exon 2 in about 1 out of 3 transcripts in WT. In contrast, this exon skipped isoform is rarely detected in *clcn7^−/−^* (1 in 60 transcripts) (Supplementary Fig. 1b). Taken together with the undetectable bone resorption in scales, this is consistent with the mutation creating a loss-of-function allele.

Differential gene expression analysis of these data detected 22,459 expressed genes with 1,900 significantly upregulated (log_2_fold ≥ 1, *P*adj < 0.05) and 849 significantly downregulated (log_2_fold ≤ −1, *P*adj < 0.05) in *clcn7^−/−^* compared with WT (Fig. 4a; Supplementary File 1). Interestingly, *clcn7* transcript abundance did not decrease in *clcn7^−/−^* (log_2_fold = 0.22, *P*adj = 0.5465), indicating that the frameshift and premature termination codon did not trigger nonsense-mediated mRNA decay (Table 1). However, the expression of other genes involved in bone resorption that when altered also cause osteopetrosis in humans, such as *tcirg1b, snx10b*, and the CLC-7 beta-subunit *ostm1*, significantly increased in *clcn7^−/−^* (Fig. 4b and Table 1) (Palagano et al. 2018; Funck-Brentano et al. 2024). Similarly, gene set enrichment analysis showed an enrichment in genes related to ATPase activity in *clcn7^−/−^* osteoclasts (Fig. 4c). Accordingly, many genes encoding subunits of the V-ATPase proton pump, which works in concert with CLC-7 to acidify resorption lacunae, had increased expression in response to loss of CLC-7 function (Supplementary File 1) (Chen et al. 2026). The broad upregulation of components of the resorptive machinery could be a compensatory response to the lack of resorptive activity and feedback signaling consistent with the increase in bone.

**Fig. 4.**
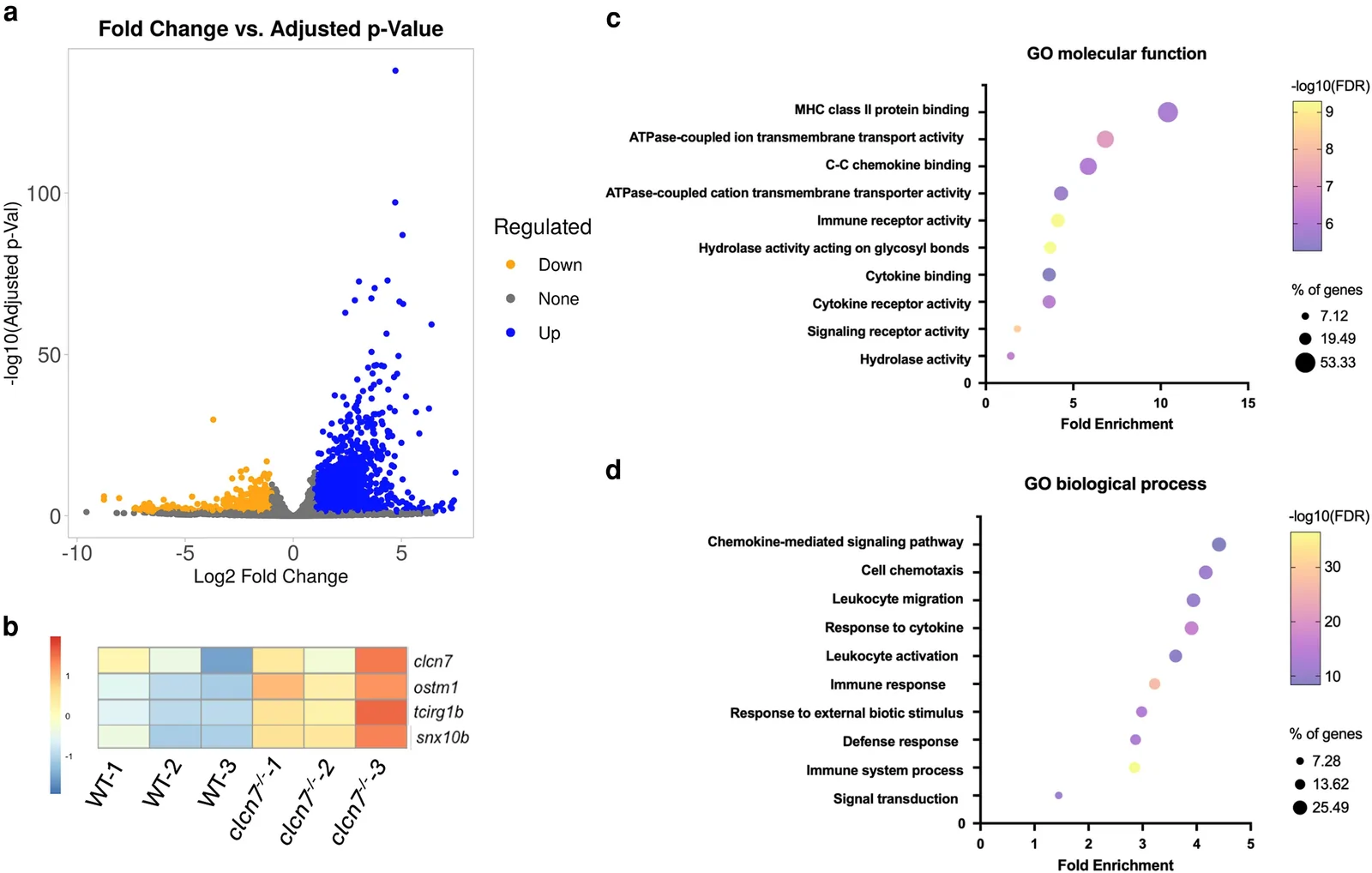
Transcriptome analysis of the effect of *clcn7* deficiency on zebrafish primary osteoclasts. a) Volcano plot showing genes differentially expressed between *dsRed*-positive cells isolated from WT and *clcn7* mutants. Blue dots represent the 1,900 genes significantly upregulated (log_2_fold ≥ 1, *P*adj < 0.05) in *clcn7^−/−^* osteoclasts. Orange dots highlight the 850 genes significantly downregulated (log_2_fold ≤ −1, *P*adj < 0.05). b) Heat map of gene expression levels of *clcn7*, and other genes known to cause ARO when mutated. Expression levels are normalized in each row. High expression is marked in red, and low levels in blue. c, d) Gene set enrichment analysis for terms related to molecular function c) and biological processes d) show the top 10 most differentially regulated pathways. The size of the dots indicates the number of genes within each pathway that are differentially regulated out of the expressed genes in that pathway. Dot color indicates the −log10 of the false discovery rate (FDR).

**Table 1.** Differential expression of genes known to cause osteoclast-rich osteopetrosis when altered.

Gene symbol	Gene name	Log_2_ fold change	*P*adj
*clcn7*	*chloride voltage-gated channel 7*	0.22	0.5465
*ostm1*	*osteoclastogenesis associated transmembrane protein 1*	1.30	6.70 × 10^−6^
*tcirg1b*	*T cell immune regulator 1, ATPase H + transporting V0 subunit a3b*	2.32	9.04 × 10^−9^
*snx10b*	*sorting nexin 10b*	1.73	0.0002

Listed are the gene symbols, gene names, log_2_ fold change, and the adjusted *P*-value (*P*adj) of genes that are differentially regulated in *clcn7^−/−^* osteoclasts and that are known to cause osteoclast-rich osteopetrosis when mutated.

Other gene sets that were found to be enriched in *clcn7^−/−^* osteoclasts included chemokine and cytokine signaling and cell chemotaxis (Fig. 4d; Supplementary File 1). Among genes that had previously been suggested to function as clastokines, only *pdgfba* and *sclerostin* (*sost*) were differentially expressed (Supplementary Table 3) (Kusu et al. 2003; Sanchez-Fernandez et al. 2008). *Pdgfba* expression was significantly increased in *clcn7^−/−^*, while expression of the inhibitory factor *sost* was significantly reduced, although < 2-fold.

As we found an increase in bone thickness and bone formation in *clcn7^−/−^* fish, we reasoned that *clcn7^−/−^* osteoclasts would secrete increased levels of anabolic factors, or clastokines. Among the 1,900 genes whose expression significantly increased in *clcn7^−/−^* osteoclasts, we narrowed down this list by identifying known secreted factors using the predicted human and zebrafish matrisome as a reference (Hynes and Naba 2012; Nauroy et al. 2018). Of the 369 factors annotated as matrisome-associated secreted factors, 271 were expressed and 20 were significantly upregulated in *clcn7-*deficient osteoclasts (Supplementary File 1). Literature reviews for the 100 most significantly upregulated genes in *clcn7^−/−^* osteoclasts, yielded 6 additional candidates. Out of those 26 genes, we selected the 9 most significantly upregulated genes to test their effect on bone development and homeostasis through transgenic overexpression (Table 2). The selected genes included 6 cytokines (*cxcl19*, *ccl34b.8*, *ccl35.1*, *M17*, *csf3a*, and *csf3b*), 1 chemotactic factor (*lect2 l*), a glycosylated peptide (*grn1*), and a lectin (*lgals9l1*). Out of the overexpressed *grn* paralogues (*grn1*, *grn2*, and *grna*), we picked *grn1* due to its small size to facilitate cloning. We excluded *tnfb*, *tgfb1b*, *il1b*, and *angpt2a* for their known pro-inflammatory and angiogenic functions. For ubiquitous overexpression, the ORFs of the selected genes were cloned into a Tol2 transgenesis vector, downstream of the *ubiquitin b* (*ubi*) promoter, and injected into WT embryos (Fig. 5a). We were able to generate viable, transgenic ubiquitous overexpression lines for 6 of these 9 genes (Table 2).

**Fig. 5.**
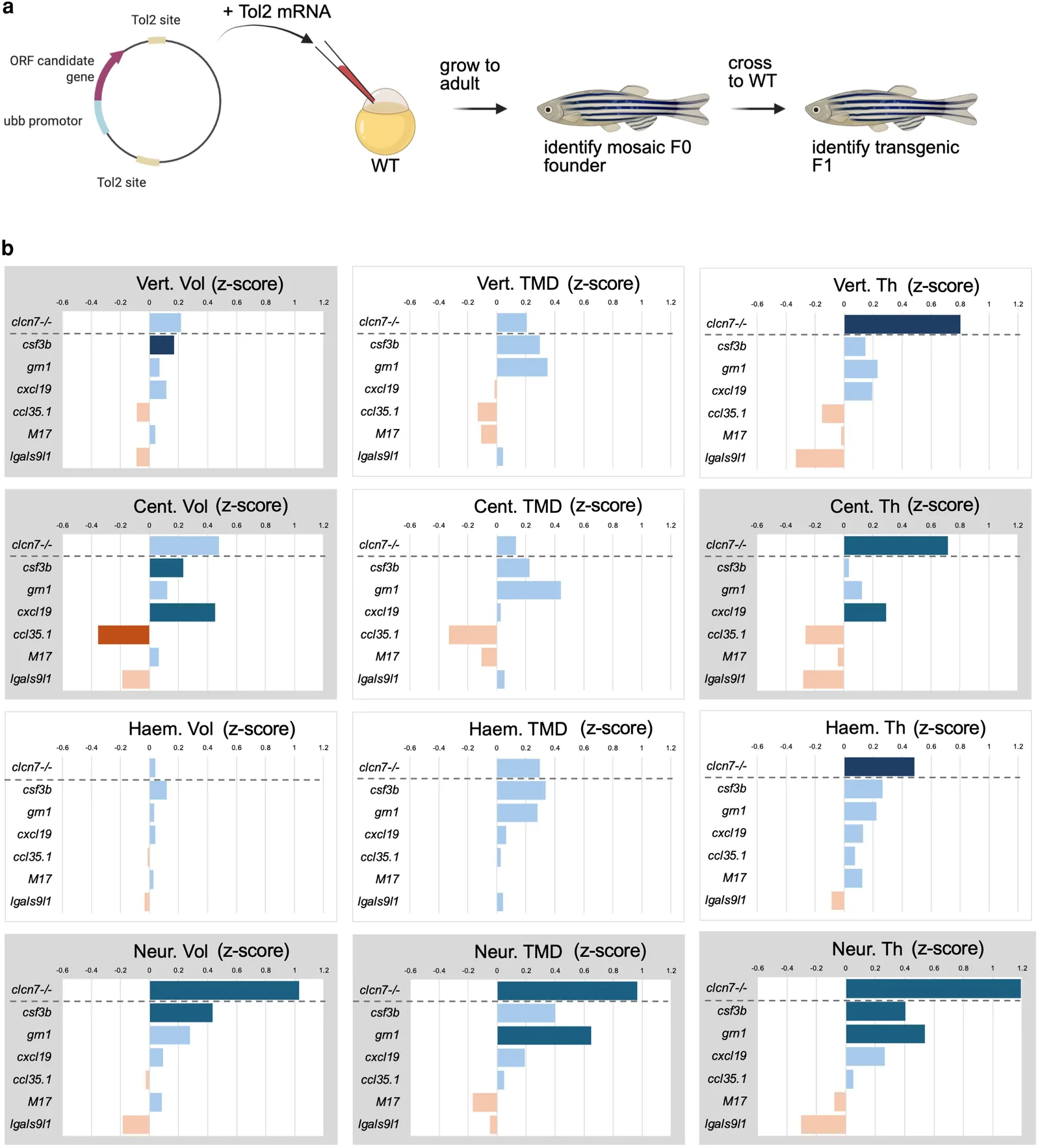
Ectopic overexpression screen identifies anabolic effects of secreted osteoclast genes. a) ORFs of selected genes were cloned downstream of the *ubi* promoter into a Tol2 transgenesis plasmid and injected together with Tol2 mRNA into fertilized WT eggs. Injected eggs were raised to adulthood, and fish with mosaic integration of the plasmid into the germline were identified (mosaic F0 founder). F0 founders were crossed to WT, and progeny were raised to adulthood. Transgenic F1 carriers were identified through genotyping and crossed to WT to establish stable transgenic lines (created in BioRender. Henke, K. (2026) https://BioRender.com/2usvck2). b) Summary data for micro-CT analysis of the vertebral column of generated overexpression lines at 3 mpf. The volume (Vol), TMD, and thickness (Th) of the whole vertebra (Vert.), as well as the centrum (Cent.), haemal arch (Haem.), and neural arch (Neur.) were analyzed separately. WT data were normalized using allometric normalization (see *Materials and Methods*). For each measure analyzed, *z*-scores highlighting the differences in value between overexpression line and sibling control are plotted. *z*-Scores for *clcn7^−/−^* fish are shown for comparison. *z*-score = (mean mutant or overexpression line value − mean control value)/SD of controls. Blue bars indicate significantly increased values compared with controls, and orange bars indicate significantly decreased values (see text for *P* values). Measures that were found to be significantly altered in at least one of the overexpression lines are highlighted through gray boxes. *n* = 10 fish/group.

**Table 2.** Secreted factors differentially expressed in WT and *clcn7^−/−^* osteoclasts selected for overexpression analysis.

Gene symbol	Gene name	log_2_fold	*P*adj	Human ortholog (DIOPT score)
***cxcl19*** ^a^	***chemokine (C-X-C motif) ligand 19***	**6**.**39**	**4.97** × 10^−60^	***CXCL8 (5)*** ^a^
*ccl34b.8^a^*	*chemokine (C-C motif) ligand 34b, duplicate 8*	3.25	1.58 × 10^−14^	*CCL19 (2)* ^a^
***ccl35.1*** *^a^*	***chemokine (C-C motif) ligand 35, duplicate 1***	**2**.**96**	**5.08** × 10^−17^	***CCL3 (10)*** ^a^
***M17***	***IL-6 subfamily cytokine M17***	**3**.**60**	**3.16** × 10^−40^	***CNTF (2)***
*csf3a*	*colony stimulating factor 3 a*	4.36	1.18 × 10^−73^	*CSF3 (3)*
***csf3b***	***colony stimulating factor 3 b***	**3**.**69**	**8.99** × 10^−24^	***CSF3 (3)***
*lect2l*	*leukocyte cell derived chemotaxin 2, tandem duplicate 1*	3.04	2.39 × 10^−73^	*LECT2 (17)*
***grn1***	***granulin a, tandem duplicate 1***	**5**.**06**	**4.91** × 10^−12^	***GRN (5)***
***lgals9l1***	***lectin, galactoside-binding, soluble, 9 (galectin 9)-like 1***	**4**.**66**	**1.00** × 10^−43^	***LGALS9 (14)***

Listed are the gene symbols and gene names of secreted factors selected for overexpression analysis, the log_2_ fold change (log2fold), and the adjusted *P* value (*P*adj). The last column shows the human homologs as identified through DIOPT, together with the DIOPT score (max. 19) in brackets.

^a^Previous analysis of chemokine genes in 18 vertebrate species has classified these genes into separate groups without direct human orthologs (Nomiyama et al. 2013). Genes for which transgenic lines were successfully established are highlighted in bold letters.

All 6 overexpression lines were fertile and without overt morphological changes. We analyzed the skeletons of 3-months-old overexpression fish and their siblings by micro-CT. Overexpression of *csf3b*, a member of the IL-6 superfamily, significantly increased neural arch thickness and volume (Neur. Th, *P* = 0.017, Neur. Vol, *P* = 0.016) as well as increased centrum volume (Cent. Vol, *P* = 0.043), which also resulted in an overall vertebral volume increase (Vert. Vol, *P* = 0.046) (Fig. 5b; Supplementary Fig. 2). Overexpression of the glycosylated peptide precursor *grn1* increased neural arch TMD and thickness (Neur. TMD, *P* = 0.043; Neur. Th, *P* = 0.003) but did not affect overall vertebral volume (Fig. 5b; Supplementary Fig. 3). The effect of *cxcl19* chemokine overexpression mainly manifested in the vertebral centrum and caused a significant increase in centrum volume and thickness (Cent. Vol, *P* = 0.039, Cent. Th, *P* = 0.009) without affecting the neural arch (Fig. 5b; Supplementary Fig. 4). Overexpression of the chemokine *ccl35.1* decreased centrum volume and thickness (Cent. Vol, *P* = 0.021, Cent. Th, *P* = 0.061), although the decrease in thickness did not reach significance (Fig. 5b; Supplementary Fig. 5). Another IL-6 superfamily member, *M17*, and the galectin *lgals9l1*, had no effect on vertebral micro-CT measures when overexpressed (Fig. 5b; Supplementary Figs. 6 and 7).

## Discussion

Recent work has shown that osteoclasts do more than resorb bone. They also express paracrine factors (clastokines) that regulate anabolic osteoblast activity (Daponte et al. 2024). Work performed on mouse models of osteopetrosis suggested that the observed increase in bone volume in these mutants was not solely due to a lack of bone resorption but was in part the result of the release of anabolic factors from osteoclasts (Thudium et al. 2014). To search for new clastokines that promote bone formation, we generated a zebrafish osteopetrosis model. Fish with an inactivating mutation in *clcn7* produce osteoclasts that are unable to resorb bone (Fig. 1). However, the fish survive to adulthood with mild but significant differences in vertebral bone shape and density compared with WT siblings (Fig. 2). This contrasts with the severe phenotypes found in mammals lacking CLC-7 function (Kornak et al. 2001). Mutations in other genes associated with early lethal forms of osteopetrosis in mammals, such as *Colony-stimulating factor 1 receptor* (*Csf1r*), also have milder phenotypes in zebrafish (Dai et al. 2002; Caetano-Lopes et al. 2020). The difference in phenotypic severity between zebrafish and mammals could be attributed to species-specific differences in the mechanisms by which bone growth occurs. In zebrafish, fin and axial skeletal growth is predominantly intramembranous, whereas limb and axial skeletal growth is mostly endochondral in mammals (Le Pabic et al. 2022). Another difference between zebrafish and mammals is the site of adult hematopoiesis. Hematopoiesis occurs at nonskeletal sites (head kidney) in zebrafish, whereas hematopoiesis in mature mammals occurs in the bone marrow (Zapata 1979). Therefore, complications arising from reduced bone marrow space in osteopetrotic mammals do not have the same impact on viability in zebrafish. An alternative explanation for the mild phenotype could be functional compensation through other related genes. Analyses of our transcriptomic data revealed an upregulation of the chloride channel genes *clcn2b* and *tthy2* in osteoclasts isolated from *clcn7^−/−^* fish (Supplementary File 1). While the absence of detectable bone resorption in our scale assay argues against a significant functional compensation through these genes, we cannot exclude that they provide a low level of functional compensation.

The ability of *clcn7^−/−^* fish to survive into adulthood and the ease of osteoclast isolation from bones in adult tissues allowed us to identify changes in expression of potential clastokines. Through differential gene expression analysis between *clcn7^−/−^* and WT osteoclasts, we identified significant increases in abundance for 1,900 genes, some of which encode secreted proteins that could be proanabolic clastokines.

Previous work in mammals has identified more than a dozen osteoclast-derived factors proposed to have roles in osteoblast recruitment and function (Supplementary Table 3). Although many of these genes are expressed by zebrafish osteoclasts, only *pdgfba* was significantly upregulated in *clcn7^−/−^* fish. Deletion of the PDGFB homodimer PDGF-BB in TRAP positive cells of the mouse was previously found to decrease trabecular and cortical bone mass (Xie et al. 2014). Moreover, Ctsk inhibition increased endogenous PDGF-BB expression and bone formation. Additional studies are needed to determine whether zebrafish bone responds to increased *pfgfba* expression similar to mouse bone.

Our differential gene expression analysis identified many transcripts encoding chemokines that were increased in *clcn7^−/−^* osteoclasts. Chemokines play a significant role during bone remodeling as they regulate the recruitment as well as differentiation and function of both osteoclasts and osteoblasts (Brylka and Schinke 2019). Some of the upregulated chemokines may be procatabolic, since we observed more TRAP positive cells on scales of *clcn7^−/−^* fish (Fig. 1). Little is known about procatabolic chemokines in zebrafish, and the rapid evolution of chemokines makes identification of direct orthologs of human and mouse genes difficult (Nomiyama et al. 2013; Bussmann and Raz 2015). However, the increased TRAP staining may also be an indirect feedback due to sensing of increased bone formation and not directly related to these particular chemokines. Therefore, further experiments are needed to elucidate which differentially expressed genes are involved in osteoclast precursor recruitment, and the zebrafish tools described here are well suited for such future analyses.

Our greater interest in this work is in factors secreted by osteoclasts that can enhance the differentiation and function of osteoblasts. Using matrisome databases and individual gene curation, we identified 26 genes that exhibited increased expression in *clcn7^−/−^* osteoclasts and could represent proanabolic clastokines. We attempted to generate global overexpression lines for 9 of these candidates and successfully generated 6 (Table 2). The reason for failure to recover stable lines for the other 3 candidates may be that viability is not consistent with broad overexpression of these factors during development. Three of the successfully generated overexpression lines increased bone mass relative to WT, 1 decreased, and 2 had no effect. Interestingly, *csf3a* and *csf3b*, which both exhibited increased expression in *clcn7^−/−^* osteoclasts, are orthologs of human CSF3, also called granulocyte colony-stimulating factor. CSF3, a member of the IL-6 family of cytokines, plays an important role in the regulation of granulocyte differentiation and function (Mehta and Corey 2021). Transgenic overexpression of *Csf3* in mice increased osteoclast number and caused osteoporosis (Takahashi et al. 1996). In contrast, global overexpression of *csf3b* in zebrafish increased vertebral volume and neural arch thickness (Fig. 5b). While a stable overexpression line for *csf3a* could not be established, the difference in phenotype between mice overexpressing *Csf3* and zebrafish overexpressing *csf3b* could be due to an evolved new function of the *csf3b* paralog in zebrafish, or subfunctionalization of early lethal roles allowing more nuanced activities of *csf3* at later developmental time.

We created fish that globally overexpress *granulin 1* (*grn1*) and found it to be proanabolic (Fig. 5b). Granulins are secreted, glycosylated peptides produced through proteolytic cleavage of a progranulin precursor (Wang et al. 2025b). Progranulin is expressed in a variety of cell types, with high expression in myeloid cells. Granulins have cytokine-like properties and play important roles in inflammation and regulation of metabolic homeostasis. In bone, granulins were found to reduce osteoclastogenesis and promote osteoblast differentiation (Noguchi et al. 2015; Wang et al. 2025a). Interestingly, mice and human genomes contain only 1 progranulin gene, while zebrafish have 4 genes (*grna*, *grnb*, *grn1*, and *grn2*) (Cadieux et al. 2005), all of which were upregulated in *clcn7^−/−^* osteoclasts. Thus, it will be interesting to determine if other progranulins are also anabolic in zebrafish.

For *cxcl19* and *ccl35.1*, whose global overexpression increased and decreased vertebral bone properties, respectively, there are no direct human homologs (Nomiyama et al. 2013). Consequently, the relevance of these findings to mammalian bone formation is uncertain. Interesting avenues for future investigation are whether these zebrafish factors can illicit an osteoblast response in the mammalian system.

Overexpression of M17 had no effect on the formation of vertebral bones in zebrafish. Zebrafish M17 is most closely related to human ciliary neurotrophic factor (CNTF). *In vitro* experiments, as well as mouse knockouts of CNTF, suggest a sex and compartment specific role of CNTF in bone formation (McGregor et al. 2010). In female mice, the strongest anabolic effect of CNTF was on trabecular bone. Since there is little trabecular bone in zebrafish, we may be unable to detect similar anabolic effects in this species.

Our study has a few limitations. First, although Cathepsin K (Ctsk) is a lysosomal cysteine protease highly expressed in mature osteoclasts, low level expression can also be detected in other myeloid cells and osteoclast precursors. In addition, recent work has found *Ctsk* to also be expressed in other tissues, including periosteal cells (Debnath et al. 2018; Zou et al. 2022). Thus, it is possible that not all dsRed expressing cells used for RNA sequencing were mature osteoclasts. Encouragingly, genes expected to be expressed in osteoclasts, such as *acp5a*, *csf1ra*, *nfatc1*, and *tnfrsf11a*, were highly expressed in our libraries. However, we also detected expression of genes more typical for osteoblast-lineage cells, such as *bglap* and the transcription factor *sp7*. Therefore, we cannot exclude that secretion of these genes from cells other than mature osteoclasts contributed to the anabolic phenotype observed in *clcn7^−/−^* fish. Second, we did not analyze the expression pattern of candidate clastokines and used global overexpression rather than expression in mature osteoclasts to allow a more relevant assessment of potential therapeutic use. While this approach was successful in determining anabolic effects, global overexpression does not recapitulate endogenous expression levels or patterns and could produce false positives by eliciting systemic effects rather than direct regulation of osteoblast activity. Similarly, expression levels at sites of bone remodeling might be insufficient to induce a detectable anabolic response, generating false-negative results. Third, we only examined the effect of overexpressing candidate clastokines on vertebral bone parameters measured by micro-CT. Downstream experiments, such as TRAP and von Kossa staining of scales to determine osteoclast number and activity, dual calcein/alizarin labeling to measure bone growth, as well as assessment of osteoblast differentiation and proliferation dynamics, are needed to determine if these clastokines affect bone formation more broadly. Lastly, we tested only a few of the 26 candidate clastokines found to be differentially expressed.

In summary, we demonstrate the use of zebrafish deficient in osteoclast resorptive function to experimentally mine bone for potential clastokines with anabolic activity. We identified potential clastokines with increased expression in *clcn7^−/−^* osteoclasts and established a functional assay to test their anabolic effects through ubiquitous overexpression. Our work provides a foundation and new avenues for further investigation into clastokines and their molecular mechanisms of function.

## Supplementary Material

jkag221_Supplementary_Data

## Data Availability

Zebrafish strains and plasmids used to generate overexpression lines are available upon request to the corresponding author. Fastq files, count data as well as differential gene expression data of our transcriptomic analyses, are available through the GEO repository under study GSE308663. All other data are present within the article, figures, tables, and supplemental files. Log_2_ fold change, adjusted *P-*values, and read counts of significantly upregulated and downregulated genes, the top 10 differentially regulated pathways identified by GO term analyses, and a list of significantly upregulated secreted factors can be found in Supplementary File 1. Supplementary Figure legends and Supplementary Tables can be found in Supplementary File 2. Supplemental material available at G3 online.
