# Supplementary material for: In vivo screen for osteoclast derived osteogenic factors using non-resorptive osteoclasts in zebrafish bone
Source: G3 (Bethesda). 2026 Aug 20;16(10):jkag221. doi: 10.1093/g3journal/jkag221 (PMC13643786; doi:10.1093/g3journal/jkag221)
Supplement: Supplementary file 1 [file jkag221_supplementary_data.zip › Supplemental_File_S2.pdf]

Supplemental Material

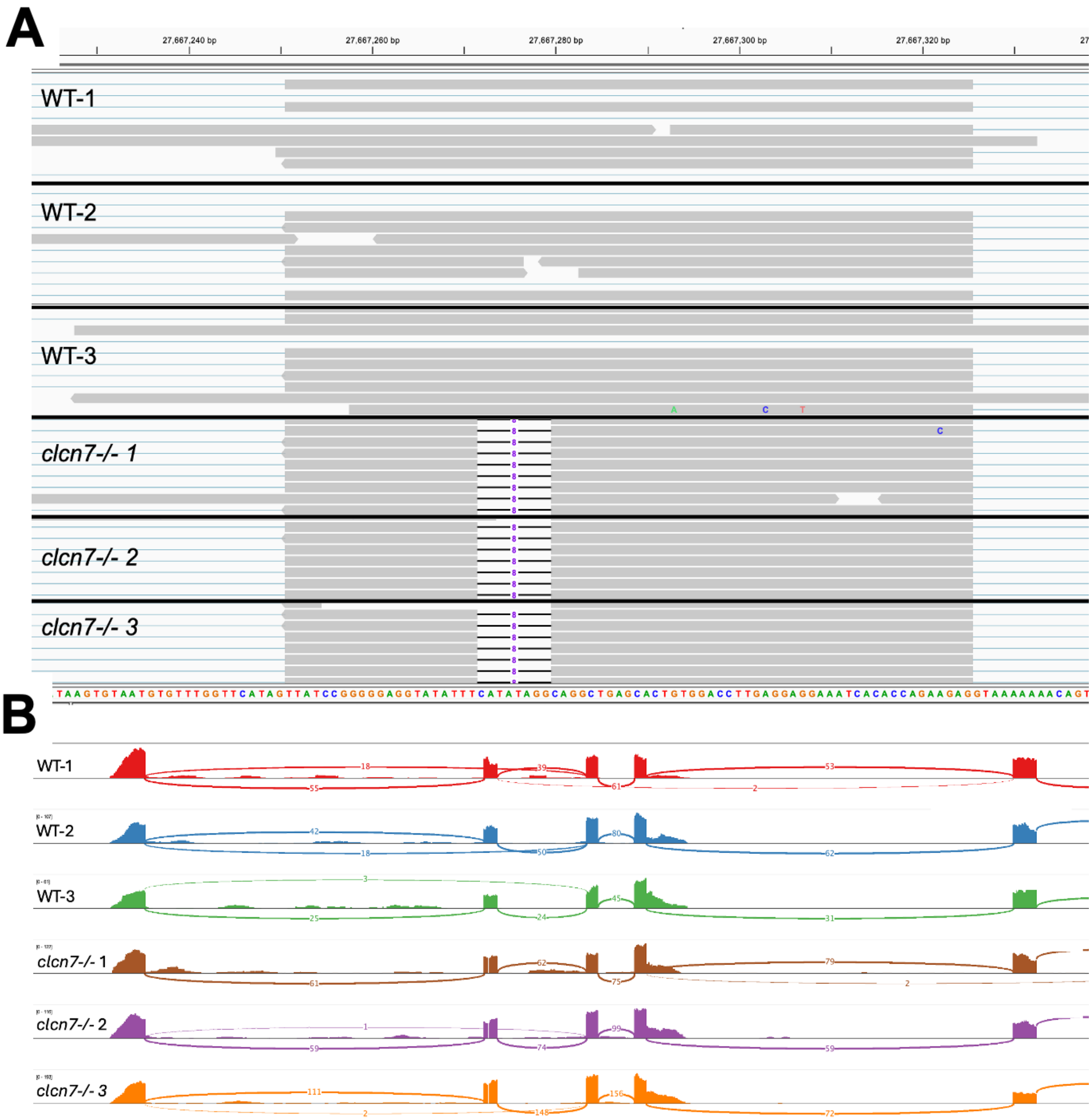

Supplementary Figure 1. Sequencing reads aligned to the GRCzv11 assembly of the zebrafish genome at the *clcn7* locus. (**A**) Alignment of sequencing reads from WT and *clcn7-/-* pools to exon 2 of the *clcn7* gene on chromosome 3. Filled grey arrowed bars indicate sequencing reads. Black lines with the purple number eight in *clcn7* pools indicate the 8 bp deletion generated through CRISPR. (**B**) Sashimi blots

showing splicing events at the first 5 exons of *clcn7*. Height of colored columns indicates sequencing depth. Numbers next to connecting lines indicate detected splicing events.

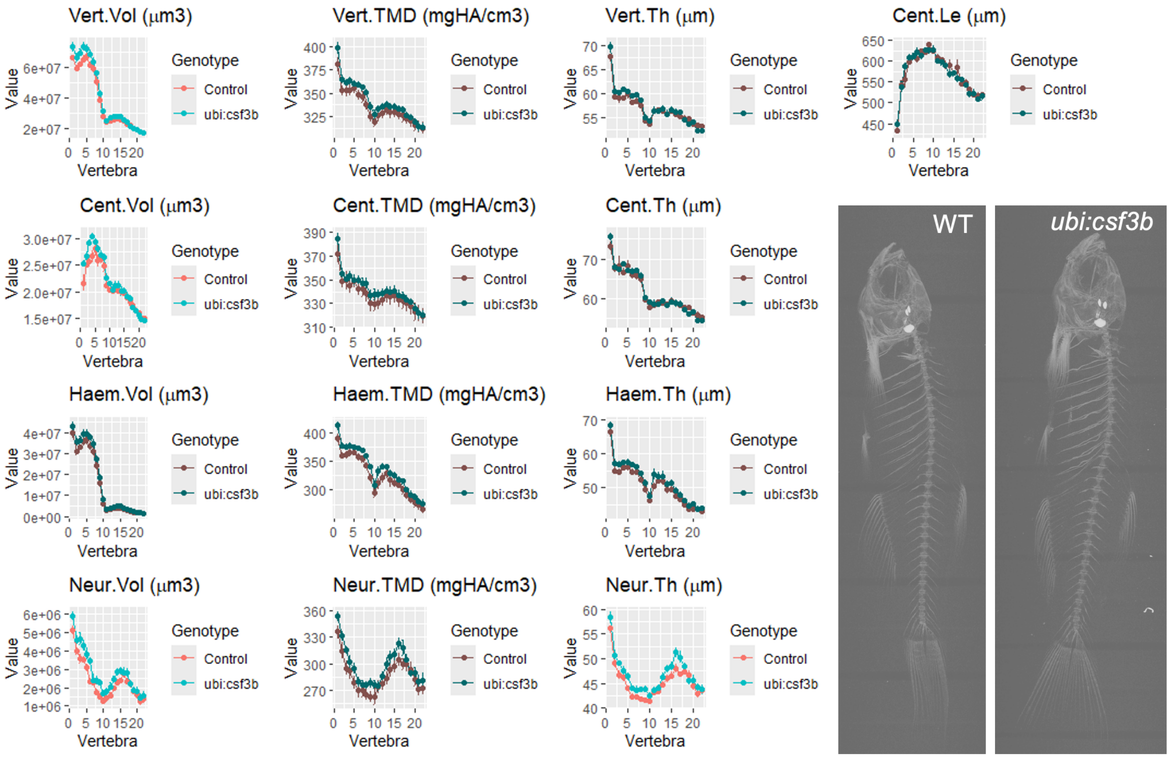

Supplementary Figure 2. Overexpression of *csf3b* increases vertebral volume as well as neural arch thickness. Micro-CT analysis of the vertebral column of 3 mpf WT and transgenic fish overexpressing *csf3b* ubiquitously. The volume (Vol), tissue mineral density (TMD) and thickness (Th) of the whole vertebra (Vert.) as well as values for the centrum (Cent.), haemal arch (Haem.) and neural arch (Neur.) separately are shown. WT data was normalized using allometric normalization. Values for abdominal and caudal vertebrae (22 total) are plotted as mean +/- SEM. Measures that were significantly different between WT and *csf3b* overexpression fish based on the global test are highlighted through lighter coloring (see text for p values). n = 10/group. Bottom right corner: representative maximum intensity projection of micro-CT scans from WT and *csf3b* overexpression fish.

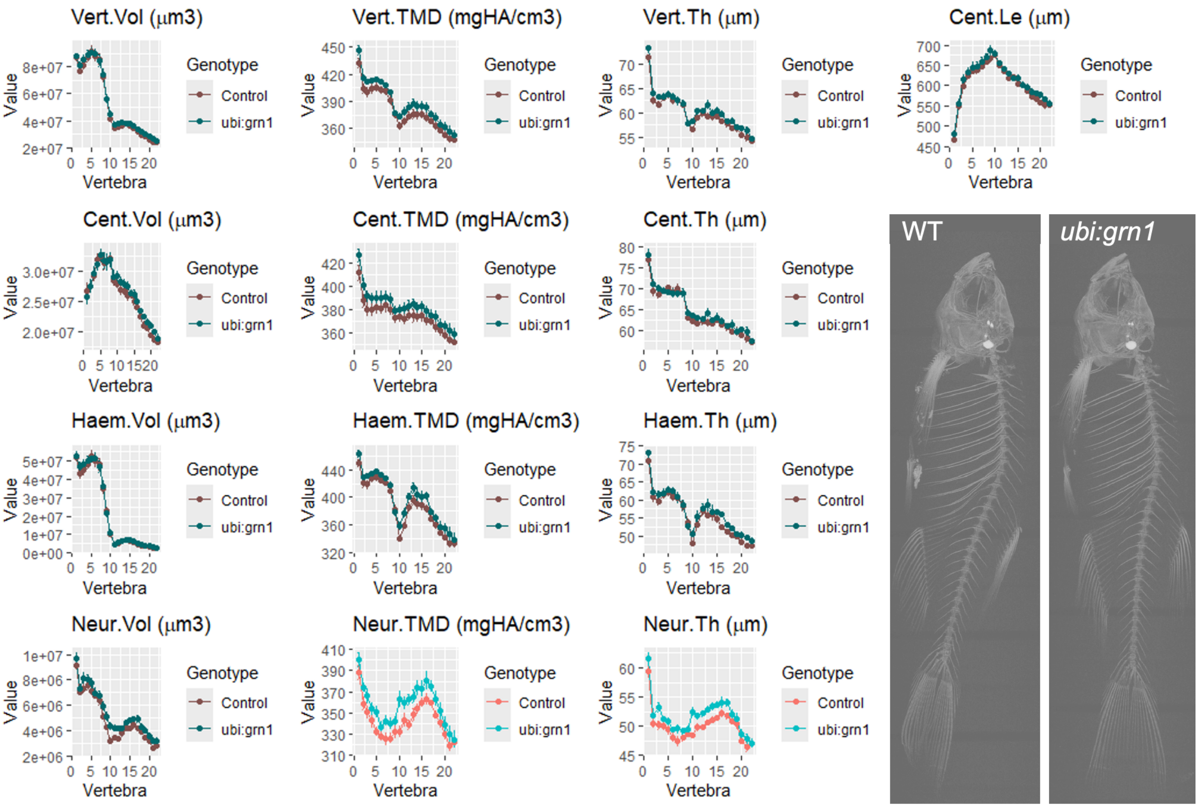

Supplementary Figure 3. Overexpression of *grn1* increases TMD as well as thickness of the neural arches. Micro-CT analysis of the vertebral column of 3 mpf WT and transgenic fish overexpressing *grn1* ubiquitously. The volume (Vol), tissue mineral density (TMD) and thickness (Th) of the whole vertebra (Vert.) as well as values for the centrum (Cent.), haemal arch (Haem.) and neural arch (Neur.) separately are shown. WT data was normalized using allometric normalization. Values for abdominal and caudal vertebrae (22 total) are plotted as mean +/- SEM. Measures that were significantly different between WT and *grn1* overexpression fish based on the global test are highlighted through lighter coloring (see text for p values). n = 10/group. Bottom right corner: representative maximum intensity projection of micro-CT scans from WT and *grn1* overexpression fish.

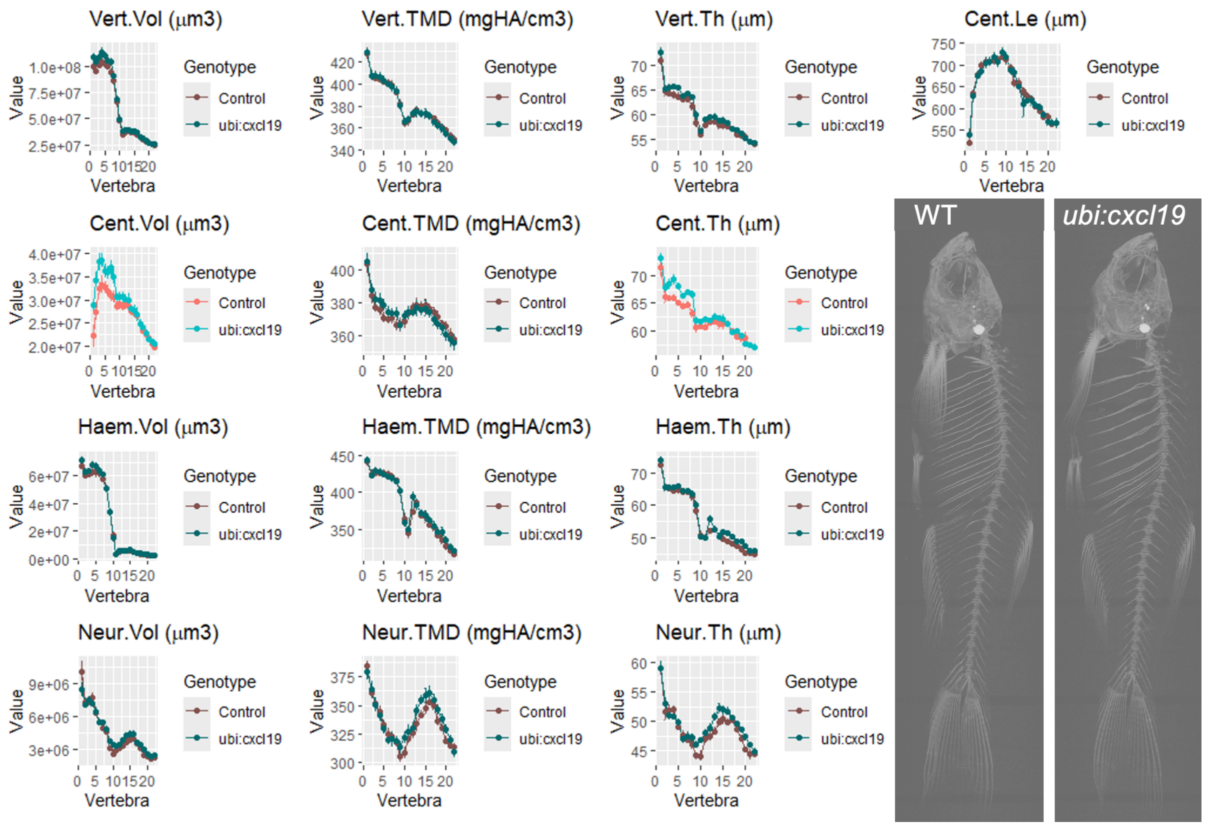

Supplementary Figure 4. Overexpression of *cxcl19* increases centrum volume as well as thickness. Micro-CT analysis of the vertebral column of 3 mpf WT and transgenic fish overexpressing *cxcl19* ubiquitously. The volume (Vol), tissue mineral density (TMD) and thickness (Th) of the whole vertebra (Vert.) as well as values for the centrum (Cent.), haemal arch (Haem.) and neural arch (Neur.) separately are shown. WT data was normalized using allometric normalization. Values for abdominal and caudal vertebrae (22 total) are plotted as mean +/- SEM. Measures that were significantly different between WT and *cxcl19* overexpression fish based on the global test are highlighted through lighter coloring (see text for p values). n = 10/group. Bottom right corner: representative maximum intensity projection of micro-CT scans from WT and *cxcl19* overexpression fish.

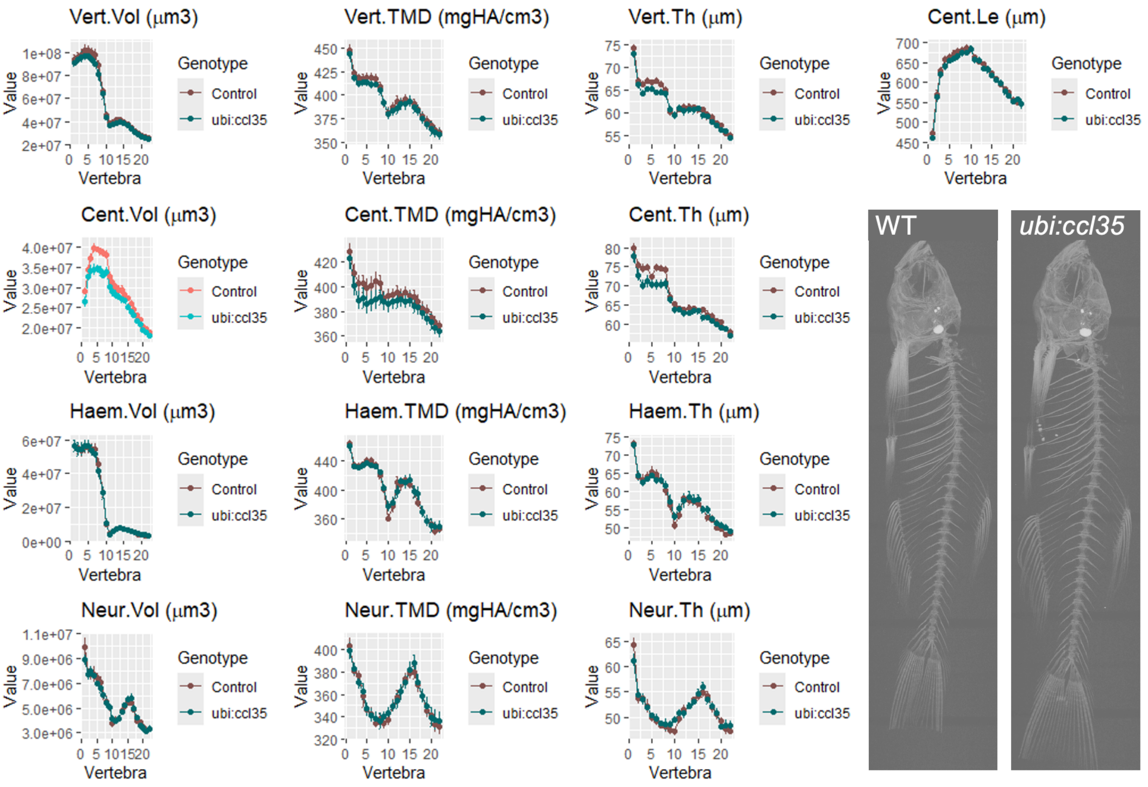

Supplementary Figure 5. Overexpression of *ccl35.1* has a negative impact on the centrum volume. Micro-CT analysis of the vertebral column of 3 mpf WT and transgenic fish overexpressing *ccl35.1* ubiquitously. The volume (Vol), tissue mineral density (TMD) and thickness (Th) of the whole vertebra (Vert.) as well as values for the centrum (Cent.), haemal arch (Haem.) and neural arch (Neur.) separately are shown. WT data was normalized using allometric normalization. Values for abdominal and caudal vertebrae (22 total) are plotted as mean +/- SEM. Measures that were significantly different between WT and *ccl35.1* overexpression fish based on the global test are highlighted through lighter coloring (see text for p values). n = 10/group. Bottom right corner: representative maximum intensity projection of micro-CT scans from WT and *ccl35.1* overexpression fish.

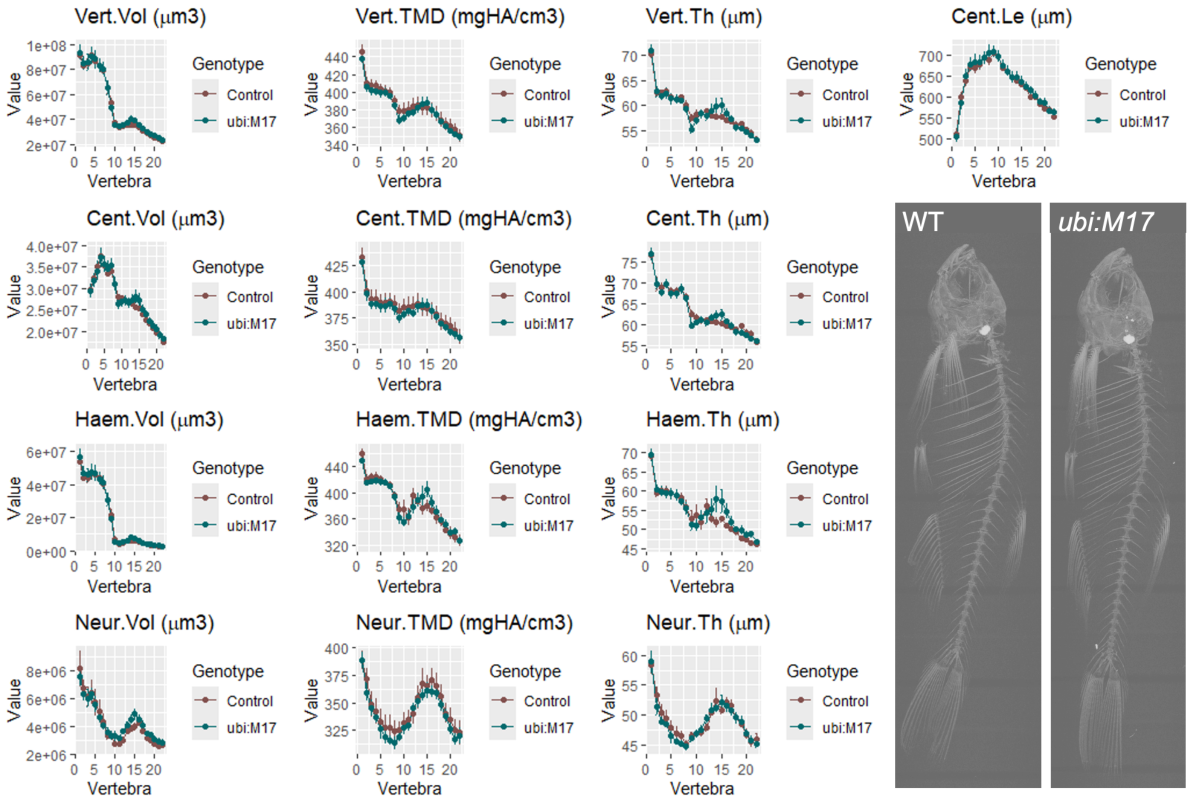

Supplementary Figure 6. Overexpression of *M17* has no significant impact on vertebrae. Micro-CT analysis of the vertebral column of 3 mpf WT and transgenic fish overexpressing *M17* ubiquitously. The volume (Vol), tissue mineral density (TMD) and thickness (Th) of the whole vertebra (Vert.) as well as values for the centrum (Cent.), haemal arch (Haem.) and neural arch (Neur.) separately are shown. WT data was normalized using allometric normalization. Values for abdominal and caudal vertebrae (22 total) are plotted as mean +/- SEM. No significant differences between WT and *M17* overexpression fish were detected using the global test. n = 10/group. Bottom right corner: representative maximum intensity projection of micro-CT scans from WT and *M17* overexpression fish.

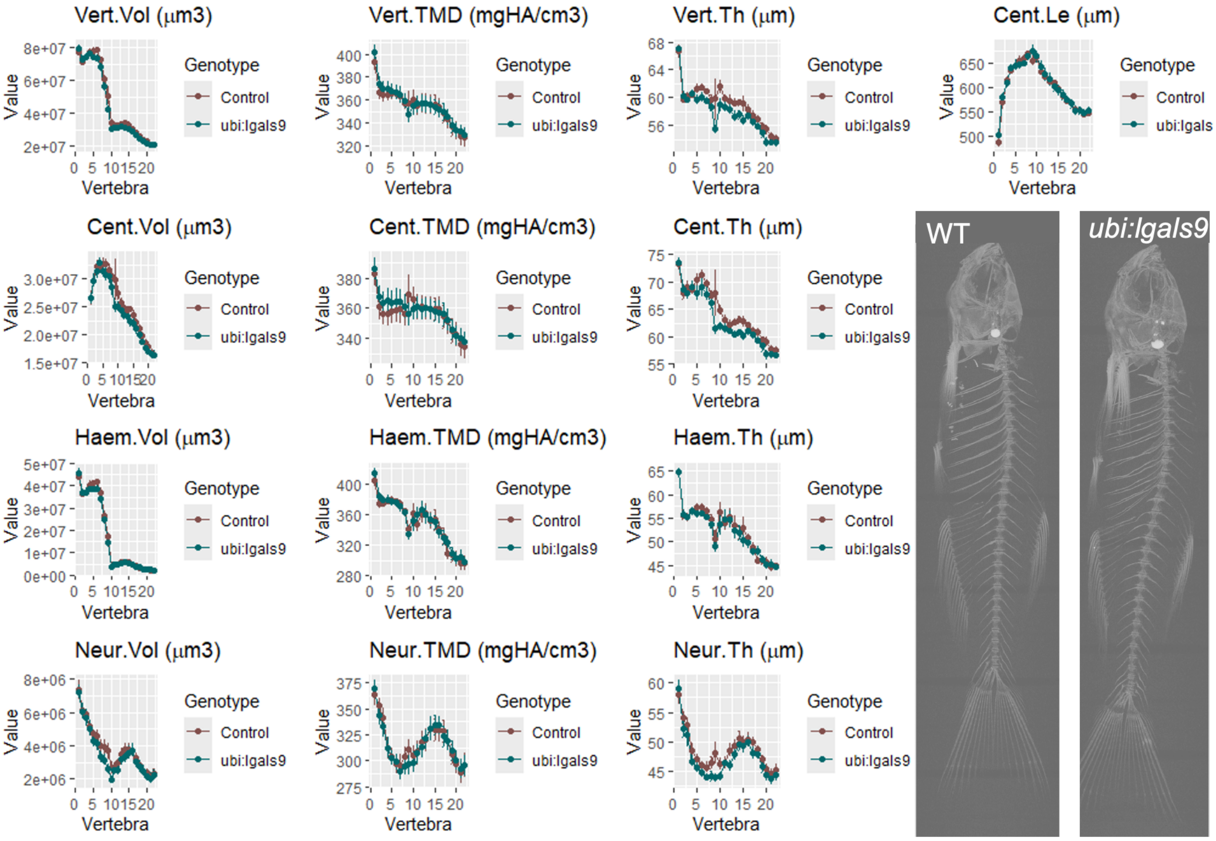

Supplementary Figure 7. Overexpression of *lgals9l1* has no significant impact on vertebrae. Micro-CT analysis of the vertebral column of 3 mpf WT and transgenic fish overexpressing *lgals9l1* ubiquitously. The volume (Vol), tissue mineral density (TMD) and thickness (Th) of the whole vertebra (Vert.) as well as values for the centrum (Cent.), haemal arch (Haem.) and neural arch (Neur.) separately are shown. WT data was normalized using allometric normalization. Values for abdominal and caudal vertebrae (22 total) are plotted as mean +/- SEM. No significant differences between WT and *lgals9l1* overexpression fish were detected using the global test. n = 10/group. Bottom right corner: representative maximum intensity projection of micro-CT scans from WT and *lgals9l* overexpression fish.

Group	Genotype	Sample Size	Average SL (cm)	SL Range
clcn7	WT	10	2.56	2.5-2.7
	clcn7$^{-/-}$	10	2.49	2.4-2.6
ccl35.1	WT	10	2.39	2.3-2.5
	ubb:ccl35.1	10	2.39	2.3-2.5
csf3b	WT	10	2.32	2.2-2.4
	ubb:csf3b	10	2.34	2.2-2.5
cxcl19	WT	10	2.51	2.4-2.6
	ubb:cxcl19	10	2.52	2.4-2.7
grn1	WT	10	2.5	2.4-2.6
	ubb:grb1	10	2.43	2.4-2.5
lgals9l	WT	10	2.41	2.2-2.5
	ubb:lgals9l	10	2.37	2.2-2.5
M17	WT	10	2.59	2.5-2.8
	ubb:M17	10	2.51	2.4-2.7

Supplementary Table 1: Standard length of fish analyzed using micro-CT. Genotypes, sample size, average standard length (SL) and SL range of groups of fish used for micro-CT analyses.

Gene name	Ensembl transcript ID	Genotyping primer for overexpression lines
ccl34b	ENSDART00000136433.2	ubb-f 5’-AAGGGTGACCAAATGCAAAG-3’<br>ccl34b-r 5’-AAGAAAAAGCAAAGCGGACA-3’
ccl35.1	ENSDART00000169041.2	ubb-f 5’-AAGGGTGACCAAATGCAAAG-3’<br>ccl35.1-r 5’-CAATGCGCATACCTTGACTG-3’
csf3a	ENSDART00000161455.3	ubb-f 5’-AAGGGTGACCAAATGCAAAG-3’<br>csf3a-r 5’-TCAAAAAGCAGAGGAGCACA-3’
csf3b	ENSDART00000165921.2	ubb-f 5’-AAGGGTGACCAAATGCAAAG-3’<br>csf3b-r 5’-AGAAGGAGGCAGCAGGTCTT-3’
cxcl19	ENSDART00000171984.3	ubb-f 5’-AAGGGTGACCAAATGCAAAG-3’<br>cxcl19-r 5’-CCAGAGTGATGCTGACTCCA-3’
grn1	ENSDART00000051972.7	ubb-f 5’-AAGGGTGACCAAATGCAAAG-3’<br>grn1-r 5’-CACAGTTTGAGCATCGCAGT-3’
lect2l	ENSDART00000032547.8	ubb-f 5’-AAGGGTGACCAAATGCAAAG-3’<br>lect2l-r 5’-CCCGAATTTCACTTGAGCAT-3’
lgals9l1	ENSDART00000100145.5	ubb-f 5’-AAGGGTGACCAAATGCAAAG-3’<br>lgals9l-r 5’-ACCTGGTAAAACCCGTCCAC-3’
m17	ENSDART00000137503.2	ubb-f 5’-AAGGGTGACCAAATGCAAAG-3’<br>m17-r 5’-TGAGCTTCAGACTTCGGTGA-3’
ostn	ENSDART00000157190.3	ubb-f 5’-AAGGGTGACCAAATGCAAAG-3’<br>ostn-r 5’-AGAGCAGCACACATCCACAG-3’

Supplementary Table 2: Genotyping primers for secreted genes selected for overexpression. Listed are the gene names and Ensembl transcript IDs of genes used in the overexpression analysis together with the primer sequences used for genotyping.

Protein symbol	Protein name	Reference	potential zebrafish orthologs	DIOPT score (max 19)	log2fold change	padj
CTHRC1	Collagen Triple Repeat Containing 1	*(Takeshita et al. 2013)*	*cthrc1a*	14	0.3021	0.521861246
			cthrc1b	17	-0.3442	0.751027095
C3a	Complement Factor 3a	*(Matsuoka et al. 2014)*	*c3a.1*	17	-0.8021	0.591757328
			c3a.2	17	-2.3357	0.28739864
			c3a.3	17	-0.5310	0.882812703
			c3a.4	17	1.4808	0.071315007
			c3a.6	14	-0.5186	0.85867872
TRAcP[1]	Tartrate Resistant Acid Phosphatase	*(Sheu et al. 2003)*	*acp5a*	17	0.4223	0.57356451
			acp5b	9	1.7986	0.163758787
BMP6	Bone Morphogenic Protein 6	*(Pederson et al. 2008)*	*bmp6*	15	0.0935	0.908629098
Wnt10b	Wingless-type MMTV integration site family, member 10b	*(Ota et al. 2013a)*	*wnt10b*	17	0.1997	0.743882746
Wnt5a	Wingless-type MMTV integration site family, member 5A	*(Roberts et al. 2020)*	*wnt5a*	11	-0.6572	0.38883206
SOST	Sclerostin	*(Kusu et al. 2003)*	*sost*	17	-0.8819	0.005607804
HGF	Hepatocyte Growth Factor	*(Grano et al. 1996)*	*hgfa*	17	0.5765	0.445690782
			hgfb	10	-0.0212	0.977435914
PDGF-BB	Platelet Derived Growth Factor B homo-dimer	*(Sanchez-Fernandez et al. 2008; Xie et al. 2014)*	*pdgfba*	6	1.4413	1.90E-06
			pdgfbb	11	-1.3537	0.08391307
SLIT3	SLIT Guidance Ligand 3	*(Kim et al. 2018)*	*slit3*	18	-0.4954	0.084042859
S1P	Sphingosine-1-Phosphate, produced by SPHK1	*(Pederson et al. 2008)*	*sphk1*	13	0.3789	0.89299805
CT-1	Cardiotrophin-1	*(Walker et al. 2008)*	*clcf1*	3	-0.5704	0.23359533
AFM	Afamin	*(Kim et al. 2012)*	*gc*	4	-1.2924	0.61793713
CXCL16	Chemokine (C-X-C motif) ligand 16	(Ota et al. 2013b)	no ortholog			
LIF	Leukemia inhibitory factor	(Koide et al. 2017)	no ortholog			

Supplementary table 3: Differential expression of proposed coupling factors. Listed are the protein symbols and protein names of proposed coupling factors, references, and gene symbols of zebrafish orthologs identified with DIOPT. The DIOPT score, which could reach a maximum of 19, is also listed for each of the potential zebrafish orthologs. The last two columns show the log2 fold-change (log2fold) detected in osteoclasts of *clcn7* mutants compared to wild type, together with the adjusted p value (padj). Significant differentially regulated genes are highlighted through grey shading.

References

Grano M, Galimi F, Zambonin G, Colucci S, Cottone E, Zallone AZ, Comoglio PM. 1996. Hepatocyte growth factor is a coupling factor for osteoclasts and osteoblasts in vitro. Proc Natl Acad Sci U S A. 93(15):7644–7648.

Kim BJ, Lee YS, Lee SY, Baek WY, Choi YJ, Moon SA, Lee SH, Kim JE, Chang EJ, Kim EY et al. 2018. Osteoclast-secreted slit3 coordinates bone resorption and formation. J Clin Invest. 128(4):1429–1441.

Kim BJ, Lee YS, Lee SY, Park SY, Dieplinger H, Ryu SH, Yea K, Choi S, Lee SH, Koh JM et al. 2012. Afamin secreted from nonresorbing osteoclasts acts as a chemokine for preosteoblasts via the akt-signaling pathway. Bone. 51(3):431–440.

Koide M, Kobayashi Y, Yamashita T, Uehara S, Nakamura M, Hiraoka BY, Ozaki Y, Iimura T, Yasuda H, Takahashi N et al. 2017. Bone formation is coupled to resorption via suppression of sclerostin expression by osteoclasts. J Bone Miner Res. 32(10):2074–2086.

Kusu N, Laurikkala J, Imanishi M, Usui H, Konishi M, Miyake A, Thesleff I, Itoh N. 2003. Sclerostin is a novel secreted osteoclast-derived bone morphogenetic protein antagonist with unique ligand specificity. J Biol Chem. 278(26):24113–24117.

Matsuoka K, Park KA, Ito M, Ikeda K, Takeshita S. 2014. Osteoclast-derived complement component 3a stimulates osteoblast differentiation. J Bone Miner Res. 29(7):1522–1530.

Ota K, Quint P, Ruan M, Pederson L, Westendorf JJ, Khosla S, Oursler MJ. 2013a. Tgf-beta induces wnt10b in osteoclasts from female mice to enhance coupling to osteoblasts. Endocrinology. 154(10):3745–3752.

Ota K, Quint P, Weivoda MM, Ruan M, Pederson L, Westendorf JJ, Khosla S, Oursler MJ. 2013b. Transforming growth factor beta 1 induces cxcl16 and leukemia inhibitory factor expression in osteoclasts to modulate migration of osteoblast progenitors. Bone. 57(1):68–75.

Pederson L, Ruan M, Westendorf JJ, Khosla S, Oursler MJ. 2008. Regulation of bone formation by osteoclasts involves wnt/bmp signaling and the chemokine sphingosine-1-phosphate. Proc Natl Acad Sci U S A. 105(52):20764–20769.

Roberts JL, Liu G, Paglia DN, Kinter CW, Fernandes LM, Lorenzo J, Hansen MF, Arif A, Drissi H. 2020. Deletion of wnt5a in osteoclasts results in bone loss through decreased bone formation. Ann N Y Acad Sci. 1463(1):45–59.

Sanchez-Fernandez MA, Gallois A, Riedl T, Jurdic P, Hoflack B. 2008. Osteoclasts control osteoblast chemotaxis via pdgf-bb/pdgf receptor beta signaling. PLoS One. 3(10):e3537.

Sheu TJ, Schwarz EM, Martinez DA, O'Keefe RJ, Rosier RN, Zuscik MJ, Puzas JE. 2003. A phage display technique identifies a novel regulator of cell differentiation. J Biol Chem. 278(1):438–443.

Takeshita S, Fumoto T, Matsuoka K, Park KA, Aburatani H, Kato S, Ito M, Ikeda K. 2013. Osteoclast-secreted cthrc1 in the coupling of bone resorption to formation. J Clin Invest. 123(9):3914–3924.

Walker EC, McGregor NE, Poulton IJ, Pompolo S, Allan EH, Quinn JM, Gillespie MT, Martin TJ, Sims NA. 2008. Cardiotrophin-1 is an osteoclast-derived stimulus of bone formation required for normal bone remodeling. J Bone Miner Res. 23(12):2025–2032.

Xie H, Cui Z, Wang L, Xia Z, Hu Y, Xian L, Li C, Xie L, Crane J, Wan M et al. 2014. Pdgf-bb secreted by preosteoclasts induces angiogenesis during coupling with osteogenesis. Nat Med. 20(11):1270–1278.
